# Adaptation and Response in Drylands: A Dryland Research Agenda and Campaign Strategy

**DOI:** 10.1111/gcb.70975

**Published:** 2026-07-15

**Authors:** Andrew F. Feldman, Sasha C. Reed, Konrad Wessels, Dennis Ojima, David J. P. Moore, William K. Smith, Niall P. Hanan, Cibele Amaral, Flurin Babst, Joel A. Biederman, Marcy Litvak, Natasha MacBean, Benjamin Poulter, Russell L. Scott, Alicja Babst‐Kostecka, Julia K. Green, Raymond F. Kokaly, Ryan Pavlick, Robert Swap, Shawn P. Serbin, Compton J. Tucker, Lixin Wang, Jennifer Watts, Alejandro Flores, James Rattling Leaf, Sativa Cruz, Nikki Tulley, Robert A. Washington‐Allen, Karen Prentice, Emily Kachergis, Julian Reyes, Jasmine Ryan, Michael D. SanClements, Henry W. Loescher, Allison K. Leidner, Tyson Swetnam

**Affiliations:** ^1^ Biospheric Sciences Laboratory NASA Goddard Space Flight Center Greenbelt Maryland USA; ^2^ Earth System Science Interdisciplinary Center University of Maryland College Park Maryland USA; ^3^ U.S. Geological Survey Southwest Biological Science Center Moab Utah USA; ^4^ Department of Geography and Geoinformation Science George Mason University Fairfax Virginia USA; ^5^ Natural Resource Ecology Laboratory Colorado State University Fort Collins Colorado USA; ^6^ School of Natural Resources and the Environment University of Arizona Tucson Arizona USA; ^7^ Jornada Basin LTER New Mexico State University Las Cruces New Mexico USA; ^8^ Cooperative Institute for Research in Environmental Sciences University of Colorado Boulder Boulder Colorado USA; ^9^ Environmental Data Science Innovation and Impact Lab University of Colorado Boulder Boulder Colorado USA; ^10^ Earth Lab University of Colorado Boulder Boulder Colorado USA; ^11^ Laboratory of Tree‐Ring Research University of Arizona Tucson Arizona USA; ^12^ Southwest Watershed Research Center USDA Agricultural Research Service Tucson Arizona USA; ^13^ Department of Biology University of New Mexico Albuquerque New Mexico USA; ^14^ Department of Geography and Environment Western University London Ontario Canada; ^15^ Department of Biology Western University London Ontario Canada; ^16^ Department of Environmental Science University of Arizona Tucson Arizona USA; ^17^ U.S. Geological Survey, Geology, Geochemistry, and Geophysics Science Center Denver Federal Center Denver Colorado USA; ^18^ Earth Sciences Division NASA Headquarters Washington DC USA; ^19^ Earth Sciences Division NASA Goddard Space Flight Center Greenbelt Maryland USA; ^20^ Department of Earth and Environmental Sciences Indiana University Indianapolis Indianapolis Indiana USA; ^21^ Woodwell Climate Research Center Falmouth Massachusetts USA; ^22^ Department of Geosciences Boise State University Boise Idaho USA; ^23^ North Central Climate Adaptation Science Center University of Colorado‐Boulder Boulder Colorado USA; ^24^ Bay Area Environmental Research Institute NASA Ames Research Center Moffett Field California USA; ^25^ Department of Agriculture, Veterinary, and Rangeland Sciences University of Nevada, Reno Reno Nevada USA; ^26^ U.S. Bureau of Land Management National Science Advisor Denver Colorado USA; ^27^ U.S. Bureau of Land Management National Assessment, Inventory, and Monitoring Coordinator Denver Colorado USA; ^28^ U.S. Bureau of Land Management Adaptive Management Lead Washington DC USA; ^29^ National Ecological Observatory Network, Battelle Research Initiatives Lead Boulder Colorado USA; ^30^ National Ecological Observatory Network, Battelle Director of Strategic Development, Environment, and Infrastructure Boulder Colorado USA; ^31^ U.S. Department of the Interior Office of Policy Analysis Washington DC USA

**Keywords:** arid, biosphere, drylands, ecology, field campaign, land management, remote sensing, research agenda

## Abstract

Dryland ecosystems cover 41% of Earth's land surfaces, account for 44% of cultivated lands and 60% of food sources, and make large contributions to the global water and carbon cycles. However, these ecosystems are experiencing unprecedented extremes including heatwaves, floods, and droughts as well as hotter temperatures and often declining water resource availability. These ecosystems are some of the most challenging to monitor given their high temporal variability with rapid response to their environment as well as vast spatial variability with intermixing of different plant species and life forms amongst bare soil coverage. The Adaptation and Response in Drylands (ARID) campaign was selected by National Aeronautics and Space Administration (NASA) as a scoping study to develop a research agenda for a dryland field campaign. Here, we detail our ARID science research agenda and implementation plan that were developed based on an extensive community engagement effort in over 160 events with over a thousand scientists, land managers, and Tribal communities between 2023 and 2024. The selected science themes cover drought and climate variability, ecosystem structure, function, and biodiversity, carbon cycle interannual variability and trends, and social ecological systems (land management and adaptation). We then detail our remote sensing, modeling, and field‐based strategies to capture high temporal and high spatial resolution processes. Finally, our implementation strategy is presented which includes focus area selections in a core intensive western U.S. domain and distributed international domains. This strategy includes our overarching guiding principles of using multi‐temporal airborne acquisitions and super sites as well as enhancing land management in co‐development with end‐user partners. While originally developed for NASA, our ARID report creates a blueprint for any future dryland field campaign, at any scale, that can be implemented widely for foundational and applied science objectives.

## Graphical abstract

The Adaptation and Response in Drylands (ARID) campaign was selected by NASA as a scoping study to develop a research agenda for a dryland field campaign. Here, we detail our ARID science research agenda and implementation plan that were developed based on an extensive community engagement effort in over 160 events with over a thousand scientists, land managers, and Tribal communities between 2023 and 2024. The graphical abstract shows ARID science themes and organization across four areas with sub‐themes labeled underneath. Now is the ideal time for a dryland‐focused field campaign. Emerging data show the critical contributions of drylands to the Earth system, as well as their responsiveness to change. Remote sensing and ground‐based tools finally have the spatial and temporal resolution needed to assess heterogeneous drylands, and these systems represent a valuable testbed for evaluating and improving the accuracy and utility of satellite data. Finally, land managers, federal agencies and other decision makers in drylands are asking for this research. They need the cross‐scale, actionable science that can inform management, adaptation, and mitigation solutions for Earth's drylands.

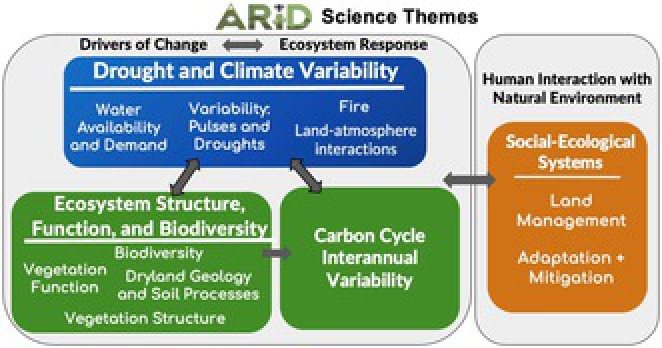

## 1 Introduction

### 1.1 Overall Importance

Drylands are defined as ecosystems with annual potential evaporative demand at least 1.5 times greater than precipitation (Wang et al. 2022). Drylands represent the planet's largest terrestrial biome, making up over 40% of Earth's land surfaces (Safriel et al. 2005). These ecosystems directly support more than 2 billion people, or about 35% of the global human population (Reynolds et al. 2007; Wang et al. 2022), and have the highest population growth rate of any ecological zone (Gaur and Squires 2018; Wang et al. 2012). Due to their vast croplands and rangelands, drylands produce 60% of the world's food supply and account for up to 44% of cultivated lands (Burrell et al. 2020; Wang et al. 2022); they therefore support livelihoods that are tightly connected to local ecosystem services. They also contain substantial mineral and energy resources, are areas of rapidly growing alternative energy installations, and support multiple dryland biodiversity hotspots around the world (Gross et al. 2024; Maestre et al. 2022). Importantly, the distinct mechanisms regulating drylands are expected to control other wetter ecosystem types, especially in a warmer and drier world (Grünzweig et al. 2022). Drylands are, therefore, of great economic, cultural, and ecological global importance.

Furthermore, although long considered an insignificant part of the global carbon cycle, in part due to misconceptions of these ecosystems as unproductive “wastelands” (Hoover et al. 2020), drylands are estimated to represent 40% of terrestrial net primary productivity (NPP), largely dominate the interannual variability (IAV) in atmospheric CO_2_ concentrations (Ahlström et al. 2015; Fan et al. 2019; Poulter et al. 2014; Sitch et al. 2024), and store a third of the planet's soil organic carbon and 79% of its soil inorganic carbon (Plaza et al. 2018).

### 1.2 Ongoing Dryland Changes

Expanding our knowledge of dryland structure and function is critical not only because of the biome's large and increasing global importance, but also because drylands are highly sensitive to extreme climate events and are responding dramatically to change. Drylands are experiencing more extreme heatwaves, droughts, and fires, putting pressure on the region's inhabitants, ecosystems, and their services (Dannenberg et al. 2022; Williams et al. 2022). Many drylands are also drying in terms of annually averaged water availability, including in the western U.S. (Zhang, Biederman, and Dannenberg 2021). Other areas are transitioning to dryland, with dryland global coverage projected to increase 11%–23% by the end of the century (Huang et al. 2016; Koppa et al. 2024). While increased evaporative demand through warming (via the Clausius‐Clapeyron relation) is causing higher specific humidity in most global ecosystems, drylands are not experiencing this higher atmospheric humidity due to already low surface soil moisture (Simpson et al. 2024). Consequently, rapid ecosystem vegetation shifts are occurring, such as mass dryland forest mortality in the face of “hot drought” (Allen et al. 2010; Anderegg et al. 2013). These effects are exacerbated by conversion of forest area to shrubland and cropland (Wang et al. 2022).

### 1.3 Extreme Event Influence on People

Effects of extreme events on drylands have been extensively highlighted by the media. In the US, the southwest experienced an extreme drought in 2020 which depleted the Colorado River flow to unprecedented lows (Fountain 2021). This extreme summer drought was a part of an ongoing multi‐decadal megadrought that started in the early 2000s and is the most extreme in this region in over one thousand years (Williams et al. 2022). This lower water availability is coupled to other events including a widespread, extreme heat wave of over 120 degrees Fahrenheit (49°C) in the western U.S. in summer 2021, responsible for over one thousand deaths (Bartusek et al. 2022). Similarly, in summer 2024, many western U.S. cities (like Phoenix, Arizona) broke records for time spent above 110 degrees Fahrenheit (43°C), also causing hundreds of fatalities (Ramirez 2024). In 2025, extreme events in dry populated areas included a devastating wildfire in populated neighborhoods of Los Angeles in January 2025 and fatal flash flooding in Texas in July 2025. While only highlighting a few of these events, it is clear that these extremes will continue to stress dryland‐based water resources, ecosystem function, and human well‐being for decades to come. Our ability to understand, monitor, forecast, and make decisions for these rapidly changing ecosystems must evolve now to keep pace with exceptional extremes and rates of change.

### 1.4 Land Management/End‐User Support

Under increasingly extreme weather and substantial change, dryland land managers are burdened with highly challenging decision making. In the western US, for example, the expansive drylands are largely managed by Federal and State agencies, Tribal nations, private corporations such as mining and cement industries, non‐governmental organizations, and private landowners. These decision makers have long had notorious difficulties managing drylands due to drylands' expansive area, high sensitivity to extremes and perturbation, low water availability, and low soil fertility. Nevertheless, these challenges present an opportunity: an improved understanding of the drivers of change, ecosystem responses, and adaptation and mitigation potential for drylands can make strides in improving natural resource management and ultimately societal outcomes.

A large focus of land management in drylands is cropland and rangeland management. In particular, rangelands constitute the world's largest land use, and the majority are in drylands (Maestre et al. 2022). Global rangelands are experiencing substantial changes to vegetation community composition and forage productivity from wildfire, weather extremes, and invasive species (including shrub encroachment and invasive grasses) (Boone et al. 2018; Godde et al. 2021), with substantial impacts on the livelihoods of more than a billion people (Maestre et al. 2022). Relatedly, there is a likelihood of potential shifts to recreational and energy‐focused rangeland uses (Briske et al. 2023) and agricultural abandonment in drylands as irrigation water becomes more limiting.

Other substantial challenges remain for decision makers in drylands. This includes managing stressed water resources under higher levels of evaporative demand, longer dry spells, and less annual rainfall (Ojima 2021; Zhang, Biederman, and Dannenberg 2021). At the same time, there is increased water use and consumption to meet food, potable water, and energy needs to support rapidly growing dryland urban centers. Others are considering drylands for their afforestation and renewable energy potential as a natural climate solution given their large land area. However, poor understanding of the dryland carbon and energy balance greatly limits the ability to quantify potential benefits under future conditions (Barron‐Gafford et al. 2016; Novick et al. 2024). For example, planting trees can increase carbon uptake, cool the surface, and improve air quality (Duveiller et al. 2018). However, these changes can have unintended consequences in some dry locations of higher wildfire risk, reduced carbon uptake benefits, and reduced vegetative cooling (Feldman et al. 2023; Rotenberg and Yakir 2010; Williams et al. 2022). Similar concerns are raised for renewable energy land uses, that while they generate energy, they also warm temperatures of the surrounding areas (Barron‐Gafford et al. 2016).

### 1.5 Monitoring Challenges and Uncertainties Needed To be Addressed

Perhaps the largest driver of poor dryland understanding is that drylands are challenging to monitor because they vary greatly in vegetation and soil composition within and across ecosystems. They span hyperarid to subhumid ecosystems and contain vegetation types from grasses and succulents to shrubs and forests amongst bare soil and biological soil crusts. They also comprise some of the most temporally variable ecosystems; they respond rapidly to rainfall pulses and exhibit large interannual variability in ecosystem processes under year‐to‐year variations of water, light, and temperature. This has led to substantial challenges and knowledge gaps. For example, dryland water and carbon fluxes and stocks are some of the most uncertain of any ecosystem type (Fawcett et al. 2022). Consequently, many global scale land surface models cannot replicate the dryland net or gross carbon uptake or year‐to‐year variability observed at in situ flux tower sites (MacBean et al. 2021; Teckentrup et al. 2021; Wang et al. 2022). This lack of model representation of dryland ecosystem functioning is likely a result of most modeling activities developed using process understanding of mesic ecosystems, which often does not translate to drylands.

Overall, despite the importance of drylands and their global coverage, dryland ecosystems are typically understudied compared to other ecosystems (Bond‐Lamberty and Thomson 2010; Maestre et al. 2012; Smith et al. 2019). As an example, in October 2011, the United Nations (UN) Environmental Management Group published “Global Drylands: A UN System‐Wide Response” (United Nations 2011) that recognized that drylands have been historically neglected. It acknowledged that drylands have high value in the Earth system but have substantial and unaddressed management challenges.

Therefore, the objective of this paper is to define the key strategies of a dryland field campaign as determined during the NASA‐funded Adaptation and Response in Drylands (ARID) scoping process. ARID started as a scoping effort between 2023 and 2024 where the authors comprised a core scoping team that developed a strategy based on the scientific literature and through engagement with over one thousand scientists, decision makers, and data end users in over 160 events (Feldman, Reed, et al. 2024). In Section 2, we first provide motivation and background for a dryland field campaign (“why” a dryland campaign is necessary). Next, in Section 3, we lay out our dryland research agenda categorized within our ARID‐formulated science themes (“what” can be studied). In Section 4, we describe our dryland‐specific remote sensing, modeling, and field‐based strategies to be used in the campaign (“how” the campaign can be carried out). Finally, in Section 5, we define the campaign guiding principles and strategy and the prospective campaign domains (also “how” and “where” the campaign can be carried out).

This article summarizes contents of the ARID white paper final report, focusing on the science themes and implementation strategy. It also complements a recently published article by the same authors that covers the ARID community engagement strategy (see Feldman, Reed, et al. 2024).

## 2 The Need for a Dryland Field Campaign

Drylands present key uncertainties in estimates of ecosystem processes and state variables that can only be quantified and disentangled through coordinated multi‐scale field, airborne, satellite‐based, and modeling analysis. While other ecosystems, such as boreal and tropical forests, have received long duration coordinated research efforts, drylands have not yet received this sustained scientific attention and level of coordination. Over the past four decades, NASA's Terrestrial Ecology Field Campaigns have focused on temperate ecosystems (FIFE: First ISLSCP Field Experiment), arctic‐boreal ecosystems (BOREAS: Boreal Ecosystem‐Atmosphere Study and ABoVE: Arctic‐Boreal Vulnerability Experiment), and tropical forests (LBA: Large‐Scale Biosphere‐Atmosphere Experiment in Amazonia). These efforts have demonstrated the power of coordinated field campaigns to vastly advance our understanding of complex systems, to build long lasting collaborations, and to train the next generation of Earth system scientists. Additionally, other U.S. agencies like the Department of Energy have invested in arctic and tropical forest ecosystems through the Next Generation Ecosystem Experiments (NGEE), which have brought together a range of members of the science community. Such a scale of effort and degree of community building demonstrated in these former campaigns has not yet occurred for dryland ecosystems and represents a substantial and timely research opportunity. NASA has invested in dryland campaigns like Northern Eurasian Earth Science Partnership Initiative (NEESPI), Surface Biology and Geology High Frequency Time Series (SHIFT), Hydrologic Atmospheric Pilot EXperiment in the Sahel (HAPEX‐Sahel), and Southern African Regional Science Initiative 2000 (SAFARI‐2000). However, they were shorter in duration, more limited in spatial domain, and more limited in scope compared to field campaigns like ABoVE and LBA.

**FIGURE 1:**
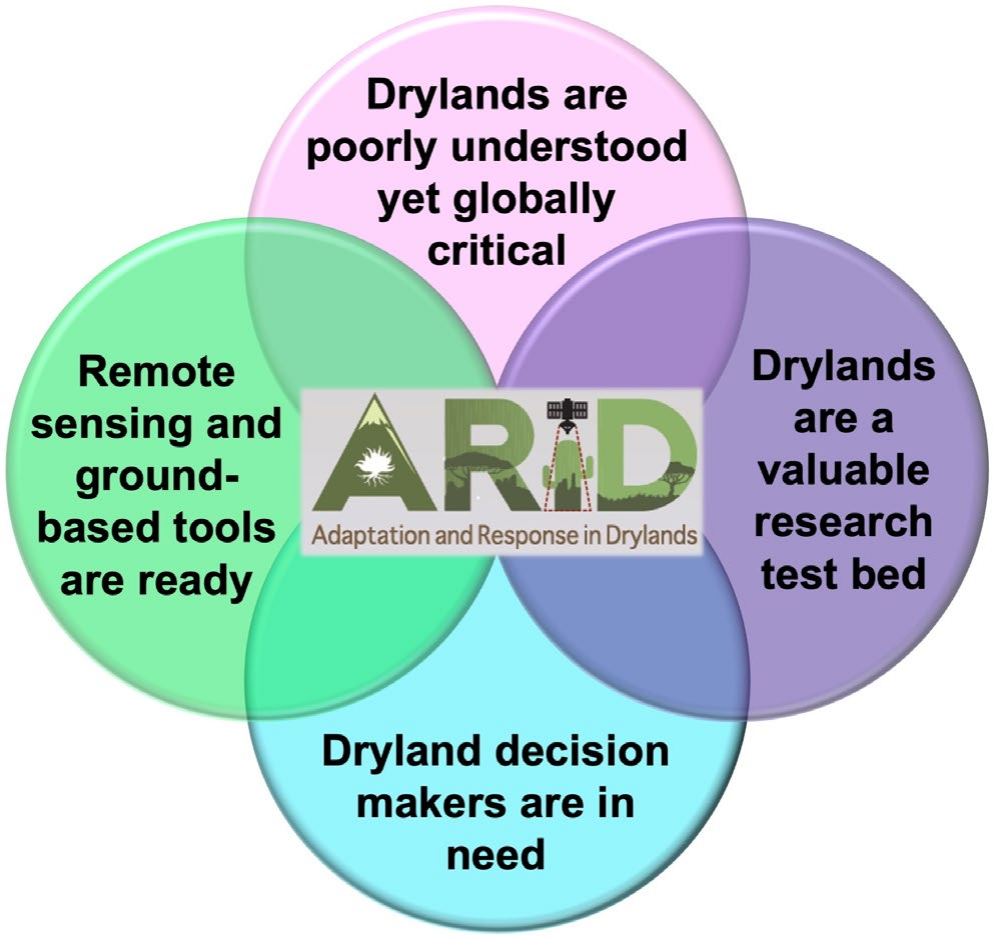
Emerging data demonstrate the major contributions of drylands to the Earth system, as well as their responsiveness to change. Remote sensing and ground‐based tools currently have the spatial and temporal resolution needed to assess heterogeneous drylands, and these systems represent a valuable testbed for evaluating and improving the accuracy and utility of satellite data. Finally, land managers, federal agencies and other decision makers in drylands can benefit from this research. They indicate that cross‐scale, actionable science could help inform management, adaptation, and mitigation solutions for Earth's drylands.

Due to drylands' high spatial and temporal heterogeneity, vast spatial expanse, and dramatic rates of change, satellite data are key to an accurate understanding of dryland functioning and trends. However, remote sensing approaches have been challenged by drylands' low vegetation signals, soil contribution to observations, and very high spatial (often meter scale) soil–plant variability and temporal variability (hourly to daily ecosystem responses). For example, estimating the fractional cover of different vegetation types in spatially heterogeneous and sparsely vegetated tree‐grass‐shrub ecosystems is challenging (Pervin et al. 2022), resulting in considerable global land surface model uncertainty in predicted carbon, water, and energy budgets (Hartley et al. 2017). Furthermore, drylands are driven by episodic precipitation pulses interspersed with extended dry periods (Collins et al. 2014; Feldman, Feng, et al. 2024; Zhang, Biederman, Pierce, et al. 2021) and experience irregular growing seasons (Cawse‐Nicholson et al. 2021). Global scale process‐based land surface models are likely not capturing such responses (MacBean et al. 2021; Teckentrup et al. 2021). Nevertheless, advances in the remote sensing, modeling, and ground‐based tools now provide more capabilities in capturing the large spatial and temporal heterogeneity in dryland systems (Figure 1). Therefore, joining satellite data with high spatial and temporal‐resolution field and airborne data within a field campaign can address the most substantial dryland research and observational gaps (Smith et al. 2019).

In addition to improving our understanding of these important water‐limited ecosystems, drylands provide a rigorous test of the ability of modern remote sensing to capture highly dynamic, spatially complex, and globally widespread ecosystems. Drylands have consistently played impactful roles in remote sensing development and evaluation (Smith et al. 2019). Due to lower cloud cover and higher likelihoods of favorable atmospheric conditions, many remote sensing techniques were developed in drylands, including early retrievals of surface reflectance and estimates of vegetation condition using red and near‐infrared bands, which would later become the Normalized Difference Vegetation Index (NDVI) (Tucker 1979). Increased spatial resolution and the ever‐expanding array of current and future space‐based missions in the public and commercial sectors provide ideal conditions for continued testing and innovation in drylands (Tucker et al. 2023).

## 3 Dryland Research Agenda: ARID Science Themes

**FIGURE 2:**
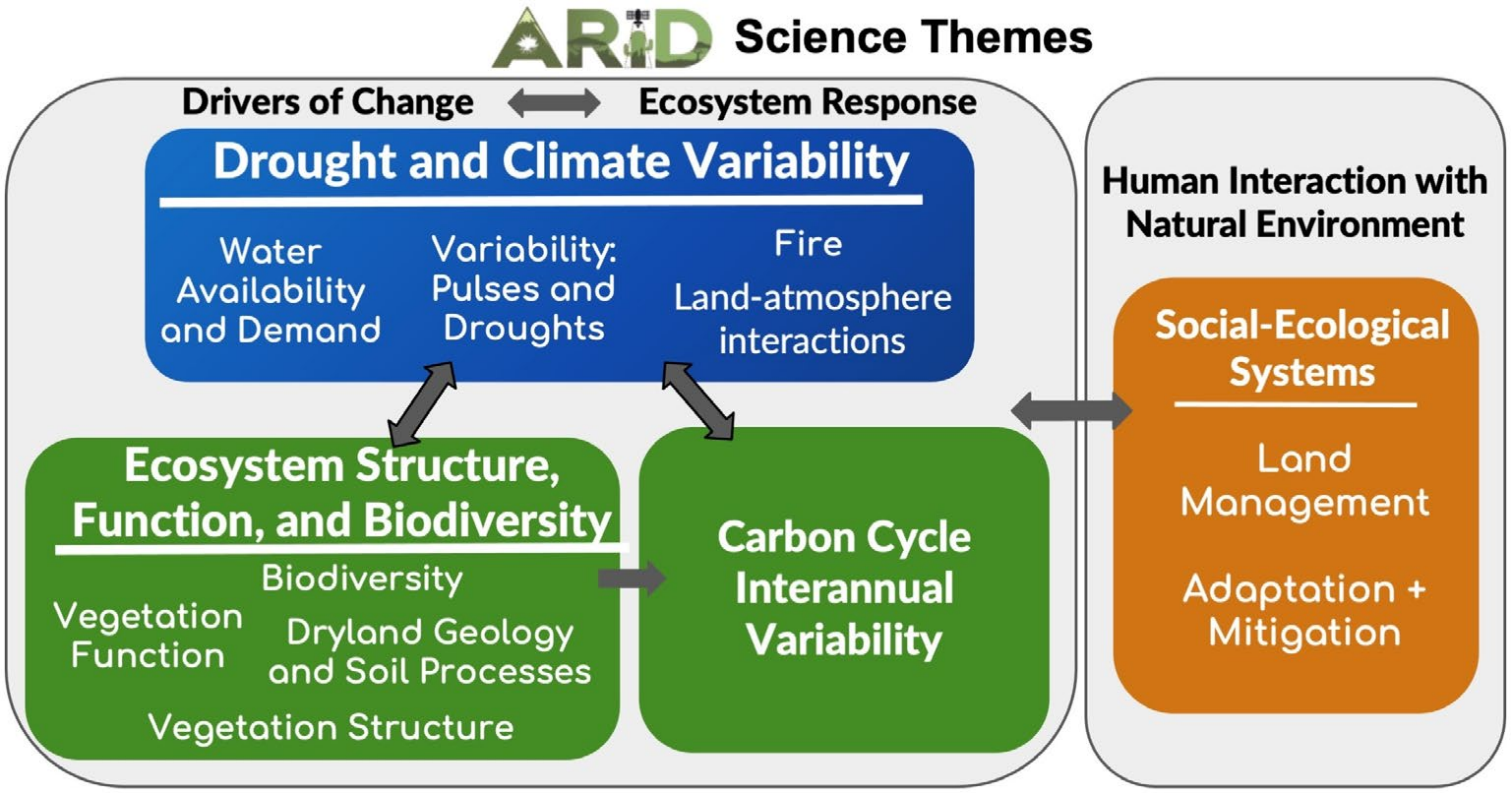
ARID science themes and organization across four areas with sub‐themes labeled underneath.

ARID's overarching science themes are Climate Variability and Drought (Section 3.1), Ecosystem Structure, Function, and Biodiversity (Section 3.2), Carbon Cycle Interannual Variability and Long‐Term Trends (Section 3.3), and Social‐Ecological Systems (Section 3.4). These themes were formulated based on extensive community engagement including input from conference townhalls, workshops, webinars, and written feedback (Feldman, Feng, et al. 2024; Feldman, Reed, et al. 2024). We discuss each theme and their subthemes (Figure 2). Together, these themes encapsulate the community's suggestions of the most pressing research questions in drylands, and lay out a dryland research agenda for the coming years. These themes are components of an integrated system linking drivers of change to ecosystem responses and human interactions (Figure 2). Importantly, all themes are interconnected through the linking of the water, carbon, and energy cycles at the surface and how human systems play a role in altering these linkages.

### 3.1 Science Theme: Climate Variability and Drought

While low precipitation levels and high evaporative demand are characteristic features of drylands, a dominant feature across global dryland regions is substantial variability driven by seasonal patterns (i.e., monsoons) and longer‐term variability like the El Niño Southern Oscillation (ENSO), the Pacific Decadal Oscillation (PDO), and the North Atlantic Oscillation (NAO) (Holmgren et al. 2006). These climate variations are responsible, for example, for fire regimes in the southwestern U.S. (Swetnam and Betancourt 1990), drought occurrence, and long‐term vegetation states (Holmgren et al. 2001). These atmosphere and rainfall drivers are leading to years with reduced water availability and increased drought impacts resulting in economic losses, altered levels of ecosystem services, damage to natural resources, and reduced capacity to maintain livelihoods. With changes to these variability processes, dryland regions are facing more extreme climate variations that further exacerbate surface conditions, including more frequent droughts and heatwaves, pulses of extreme rainfall events, increased frequency and intensity of fire, and sustained, stronger extremes through land‐atmosphere interactions.

This ARID science theme investigates how and to what extent climate extremes—like droughts, heatwaves, and large rain pulses—affect dryland systems and how they interact with land management practice and decision making, changing fire regimes, land cover change, and land‐atmosphere interactions. The main science question this theme aims to address is: How are climate extremes like droughts, heatwaves, and large rain pulses affecting dryland systems and how do they interact with changing fire regimes, land cover change, and land‐atmosphere interactions?

#### 3.1.1 Water Availability and Demand

Water is the key limiting factor for ecosystems and societies in drylands. Quantifying how water availability is changing is critical to understand and predict how dryland ecosystem structure and function will be altered in the future. This understanding is essential to inform decision makers of how to adapt management practices to conserve and enhance dryland resources. Furthermore, dryland water availability is controlled by the amount, timing, and fate of water inputs at the land surface. This includes rainfall, fog, and dew sources near large water bodies, and snowmelt at higher latitudes and/or elevations. Groundwater is also a critical source of water in some ecosystems, such as aquifer storage used for irrigation and deeper moisture sources for plant transpiration. These water sources, across streamflow, groundwater, and reservoirs, are critical for human consumptive uses. Water uses for agriculture, power generation, and mining greatly affect water availability. Water availability at the land surface is further modified by ecosystem structure, biodiversity, and functioning that control the land surface energy balance and regulate evapotranspiration (ET).

Water availability is challenging to monitor in drylands because of high spatiotemporal heterogeneity and difficulty sampling deeper soil moisture (Table 1). Furthermore, water availability is predicted to generally decrease across many drylands (Lian et al. 2021). ARID aims to address: how do changes in the amount, timing, intensity, and phase of water inputs affect surface water partitioning among evaporation, transpiration, runoff, and groundwater recharge, thereby regulating the amount of water that is available to humans and ecosystems?

**TABLE 1:** ARID research questions organized by science theme and sub‐themes.

Research theme	Theme questions	Dryland data needs and challenges	ARID campaign proposed solution
Theme 1: Climate Variability and Drought	How are extremes like droughts, heatwaves, and large rain pulses across drylands impacting water availability, and are these extremes amplified by changing fire regimes, land cover change, and land‐atmosphere interactions?		
1.1 Water Availability	How do changes in the amount, timing, intensity, and phase of water inputs affect surface water partitioning among evaporation, transpiration, runoff, and groundwater recharge in drylands, thereby regulating the amount of water that is available to humans and ecosystems?	Challenges in observing water availability across drylands' heterogeneous landscapes, including at different soil depths	Use of microwave and thermal aircraft measurements to map spatial soil moisture and evaporation variations Relating in situ measurements of soil moisture at different depths to aircraft measurements to estimate the penetration depth of microwave aircraft and satellite measurements
1.2 Dryland Climate Variability: Pulses and Droughts	How do dryland ecosystems process heterogeneous, highly dynamic moisture pulses? How does the timing and duration of lack of rainfall impact dryland function, structure, composition, and water availability? How are drought intensity, severity, and duration changing in drylands?	Lack of daily revisits needed for understanding ecosystem pulse and dry spell responses at high spatial resolution Need for better understanding of dryland evaporation and gross primary production responses to droughts and heatwaves, both in high spatial variability of response and non‐linear threshold response in time	“Pulse‐chasing” aircraft, UAS, and in situ campaigns that provide improved process understanding for benchmarking modeled responses, and for improved interpretation of geostationary satellites Repeat aircraft, unmanned aircraft system (UAS), and in situ campaigns during heatwave and drought to understand spatial variability and temporal ecosystem responses for integration into process models
1.3 Fire	How do fire regimes on rangeland, open woodland, and forested landscapes change the composition, structure, and function of drylands at various time scales? What are the fuel loads of different plant functional types (PFTs) in relation to fire risk?	Dearth of data on woody vegetation structure and its change in response to fire Lack of data on vegetation composition and its changes following fire events and fire regime change Need for fuel load estimates of woody and herbaceous fuel types	High‐resolution lidar data to measure short‐stature dryland trees and shrubs across priority landscapes. Hyperspectral imagery to map PFTs and species composition changes in response to fire events. Improve fire process representation in models for different PFTs
1.4 Land‐atmosphere Interactions	How do land‐atmosphere interactions influence climate extremes, water availability, and dryland ecosystem responses, including changes in air temperature, changes in the frequency and intensity of extreme events, as well as land use change?	Uncertain dryland water flux sensitivity to soil moisture as well as uncertain influence of fluxes and vegetation heterogeneity on humidity and temperature of the planetary boundary layer	Microwave soil moisture and hyperspectral vegetation aircraft measurements at flux tower sites to assess interactions between vegetation patterns, moisture amounts, and evaporation on the lower atmosphere
Theme 2: Ecosystem Structure, Function, and Biodiversity	What are the main mechanisms driving the spatiotemporal distributions of dryland structure, function, and biodiversity?		
2.1 Vegetation Structure and Heterogeneity	How are vegetation structure, function, and habitat in drylands responding to climatic variations (drought frequency and intensity, rainfall variability and total amounts, and fire regimes) and land use?	Vertical vegetation structure quantification is challenged by short‐statured and open canopies in drylands. High spatial heterogeneity of vegetation structure in dryland savannas and shrublands, amongst (at times) extensive bare soil cover	Using high spatial resolution aircraft flights of hyperspectral and lidar (discrete and waveform) measurements to improve spaceborne estimates of vegetation height and vertical structure products, especially for short‐stature vegetation Improved initialization of dryland vegetation type and cover in process models
2.2 Dryland Biodiversity	What are the drivers of biodiversity (functional, phylogenetic, taxonomic) in drylands, and how are these changing?	Lack of data on vegetation composition and invasive species at sufficiently high resolution	Coordinating field vegetation identifications and aircraft hyperspectral and lidar acquisitions to develop PFT maps and biodiversity metrics
2.3 Ecosystem Function	Across different timescales, what are the dominant mechanisms driving dryland function, such as plant hydraulics, leaf‐level photosynthesis, respiration, and nutrient cycling?	Lack of process models that are sufficiently resolved to represent the heterogeneity in PFTs and their stomatal and photosynthetic functioning within dryland landscapes.	Improved process‐representation of dryland plant traits in models, including their stomatal conductance and carbon assimilation sensitivity to soil moisture and rooting parameterizations
2.4 Dryland Geology and Soils	How do soil communities and physiochemical characteristics drive and respond to climate variability and ecosystem change?	Challenges in identifying and quantifying biocrust, soil type, and mineral cover	In‐situ and airborne hyperspectral measurements that enable empirical and process retrieval methods of biocrust, soil type, and mineral cover
Theme 3: Carbon Cycle Interannual Variability and Long‐Term Trends	What is the contribution of drylands to the mean, trend, and particularly the interannual variability of terrestrial carbon dynamics?		
3.1 Carbon Stocks and Fluxes	How large are the carbon stocks and fluxes in drylands, how do they vary at sub‐annual to decadal timescales, and what is their response through space and time to drivers of global change?	Need for reducing uncertainty in estimates of net carbon uptake, including annual amounts, interannual variability, and long‐term trends in drylands	Improved satellite‐based carbon flux products that quantify how vegetation remote sensing measurements (hyperspectral, thermal, and microwave) translate to carbon uptake using airborne observations over flux towers in drylands Evaluating model simulations and constraining model parameter uncertainty via data assimilation in drylands
Theme 4: Social Ecological Systems	What are the consequences of changes in drylands for social‐ecological systems and what management (e.g., mitigation and adaptation) solutions can maintain the critical services provided by drylands even in the face of change?		
4.1 Land Management	How are land and water resources and resource management being affected by drought and aridity changes?	Need for maps of invasive species, forage quality, and forage biomass to improve decision‐making	Coordinating hyperspectral and microwave radar aircraft and in‐situ measurements to develop methods to map forage biomass and invasive species from aircraft and satellites
4.2 Adaptation and Mitigation Strategies	Adaptation and resilience ○ How can dryland ecosystems enhance their resilience in the face of environmental stressors, and what adaptive strategies can local communities implement to build resilience? Mitigation options ○ How will climate variability and water limitations hinder carbon sequestration efforts?	Need for quantifying how different vegetation types alter ecosystem‐scale water use, surface temperature, and carbon uptake to inform resilience of plant communities and mitigation options	Coordinating in‐situ and aircraft observations (microwave, thermal, hyperspectral) to determine how different plant types alter soil moisture, water use, and temperature. Flight paths can be chosen over existing plots that are experimentally altering plant communities

*Note:* A full table appears in Table S1 with a breakdown of potential process questions. The right two columns show examples of dryland needs within that subtheme and potential methods that ARID can use to address these needs and challenges. The needs and solutions are examples, and an array of needs and solutions can be explored within ARID.

#### 3.1.2 Variability: Pulses and Droughts

Two substantial controls of dryland structure and function are rainfall pulse dynamics and dry spells between pulses. This includes prolonged dry spells, or droughts. Often, drought conditions co‐occur with warmer temperatures, or “hot droughts”, which can compound effects on dryland functioning (Dannenberg et al. 2022). Many dryland regions are currently experiencing more rainfall variability, with often more intense rain events and longer dry spells (Pendergrass et al. 2017). This increasing rainfall variability extends to extreme events, with more frequent and intense droughts, heatwaves, dust storms, and severe weather events.

Ultimately, the precipitation pulse dynamics of drylands are poorly understood, particularly how rain pulse inputs influence the soil‐vegetation system at hourly‐to‐daily timescales and consequently the water, carbon, energy cycles and erosion rates. A key limitation is often that sampling is too infrequent, where at least daily measurements are needed to monitor these pulse and dry spell dynamics, which drive drylands' high temporal variability (Table 1). Furthermore, terrestrial biosphere models are developed based on more humid and temperate forest ecosystems, and may have state and flux errors associated with misrepresenting pulse dynamics and drought response (Feldman, Feng, et al. 2024). ARID aims to address: How do dryland ecosystems respond to heterogeneous, highly dynamic moisture pulses as well as droughts?

#### 3.1.3 Fire

Warmer temperatures, higher evaporative demand, and declining water availability are increasing wildfire frequency and intensity across dryland ecosystems globally (Senande‐Rivera et al. 2022). Fire dynamics can also be intensified through feedbacks with biotic agents such as bark beetles that increase tree mortality and associated dead biomass fuel loads (Hicke et al. 2012). Though wildfire naturally stabilizes ecosystems, more intense and/or frequent fires can push dryland ecosystems beyond tipping points into new states (Hoover et al. 2020). For rangelands, this can result in the displacement of native species used for forage to invasive annual grass‐dominated ecosystems that further elevate fire risk (Balch et al. 2013). While fire exclusion management was largely successful for much of the 20th century, the resulting gradual accumulation of surface fuels and growth of unnaturally dense forests coupled with warming has rapidly driven increases in wildfire frequency and severity (Mueller et al. 2020). In response, a rapid paradigm shift in forest management is underway with a focus on forest restoration treatments that reduce canopy cover and fuel loads, such as mechanical thinning and prescribed fire (Fulé et al. 2012).

Multi‐scale remote sensing observations could be further integrated and coordinated with land managers to inform land management strategies toward increased forest resilience to wildfire without unintended negative consequences. ARID aims to address: How do fire regimes on rangeland, open woodland, and forested landscapes change the composition, structure, and function of drylands at various time scales?

#### 3.1.4 Land‐Atmosphere Interactions

Land‐atmosphere interactions, or the exchanges of water, carbon, and energy between the land surface and atmosphere, strongly drive dryland biogeochemical fluxes. These exchanges are particularly pronounced and coupled in drylands due to soil moisture limitations, where, for example, increased soil moisture largely increases photosynthetic functioning and evapotranspiration. The interactions can be land‐to‐atmosphere as well: rainfall can initiate over drylands because of soil moisture spatial heterogeneity and/or ET recycling (Green et al. 2017; De Kauwe et al. 2013; Koster et al. 2004; Taylor et al. 2011). These exchanges can also lead to positive feedback loops where a lack of moisture further suppresses rainfall and/or warms the surface, leading to intensified droughts and heatwaves (Schumacher et al. 2022). Indeed, low soil moisture availability can reduce terrestrial carbon uptake and prolong drought conditions (Dannenberg et al. 2022; Williams et al. 2022).

It is essential to understand these feedbacks between the land and atmosphere to accurately predict dryland climate and weather patterns as well as the associated effects on precipitation, soil moisture, ET, runoff, dust storms, and vegetation productivity and dynamics (e.g., stress response, phenology, vegetation mortality). Such processes are notably challenging to predict in drylands due to heterogeneous land cover and the strong role of water limitation on surface functioning, which are often misrepresented or biased in state‐of‐the‐art models (MacBean et al. 2021; Rogers et al. 2017). ARID aims to address: how do land‐atmosphere interactions influence and modulate climate extremes, water availability, and dryland ecosystem responses?

### 3.2 Science Theme: Ecosystem Structure, Function, and Biodiversity

Dryland landscapes are characterized by their high horizontal, vertical, and temporal heterogeneity in the cover and density of bare soil, biocrust, and distinct plant functional types (PFTs) (Smith et al. 2018). This large heterogeneity and associated high biodiversity are driven by many factors including highly variable water availability, plant facilitation, and competition for resources, and are often shaped by interactions with animals (grazing) and insects.

Drylands globally have experienced substantial changes in structure, composition, and productivity, and more substantial change is predicted under climate change and land management practices (Boone et al. 2018; Godde et al. 2021; Kleinhesselink et al. 2023). However, conventional remote sensing and modeling approaches have poorly represented vegetation structure in drylands, which are characterized by low cover and density of woody plants across spatially heterogeneous landscapes. Additionally, above‐belowground plant biomass ratios differ in drylands from those commonly observed in more humid environments. ARID aims to address: What are the main mechanisms driving the spatiotemporal distributions of dryland structure, function, and biodiversity, and what is their vulnerability to change?

#### 3.2.1 Vegetation Structure and Heterogeneity

Vegetation structure refers to the three‐dimensional spatial variation in a plant community. The fine spatial and temporal scale of dryland variability has historically meant that remote sensing and modeling of drylands has had challenges representing vegetation structure, composition, and thus function. While recent satellite and airborne remote sensing provide opportunities for substantially improved spatial and temporal resolution, improved dryland remote sensing of vegetation requires procedures for better detection and quantification of horizontal and vertical vegetation structure as well as PFT or species diversity (Table 1). Such methods will enable improved evaluation of drivers of landscape change, including how climate, land use, and fauna (e.g., American buffalo, bark beetles, etc.) shape vegetation structure and heterogeneity. This would ultimately enhance our understanding of dryland biodiversity and representation of dryland vegetation type and cover (and associated carbon, water, and ecosystem services) in Earth system models. ARID aims to address: How are ongoing changes in climatic factors (like more CO_2_, increases in drought frequency and intensity, long‐term changes in mean rainfall, and changing fire regimes) and land use (changing fire regimes, grazing, and other land uses) effecting vegetation structure, function, and habitat in drylands?

#### 3.2.2 Dryland Biodiversity

Dry ecosystems host a higher‐than‐expected functional diversity despite water resource limitations, containing 35% and 20% of global diversity and plant diversity hotspots, respectively (Maestre et al. 2021). This “functional paradox” could be attributed to unpredictable environmental conditions allowing alternative plant strategies to coexist or even facilitate each other, leading to high functional, trait, and species diversity (Gross et al. 2024). High spatiotemporal variability in water availability has driven unprecedented species diversity across global drylands, including dynamic mixtures of C3 and C4 grasses, shallow‐rooted CAM plants, deep‐rooted C3 shrub and tree species and diverse biocrust assemblages, each with specialized water acquisition strategies (Gross et al. 2024). Diverse plant communities then also support high diversity of heterotrophs across multiple trophic levels (e.g., insect and mammalian herbivores and their predators). Ultimately, this biodiversity contributes to the variability in ecosystem functions and process rates that respond to alternating drought and wet periods and other climate extremes.

The scale‐dependent processes through which structural heterogeneity and temporal variability due to disturbances (fire and rainfall pulses) drive dryland biodiversity are poorly understood. Additionally, there are several threats to dryland biodiversity loss from higher aridity, land degradation, and grazing pressure (Cartereau et al. 2023; Gross et al. 2024; Maestre et al. 2022; Montanarella et al. 2018). ARID aims to address: what are the drivers of biodiversity (functional, phylogenetic, taxonomic) in drylands, and how will these change?

#### 3.2.3 Ecosystem Function

Dryland ecosystem functioning is characterized by dormancy that endures for weeks, until a rain event recharges surface soil moisture. Depending on the timing and intensity of the rain event and edaphic factors, rain infiltration to surface and root zone soil moisture (RZSM) can drive pulses of heterotrophic respiration (Roby et al. 2022) as well as photosynthesis (gross primary productivity; GPP). Critical soil moisture thresholds otherwise limit this behavior, especially at low soil moisture (Fu et al. 2024). Much of this functioning is dictated by moisture transfer through the soil–plant‐atmosphere water continuum (SPAC); soil water supply and atmospheric demand enable root water uptake through xylem, supplying leaf water potential and transpiration. Dryland GPP is predominantly water limited and has been found to be tightly coupled with anomalies in RZSM and ET (Biederman et al. 2017; Scott et al. 2015). Slight changes in the ratios of GPP and ET, that is, ecosystem water use efficiency (WUE), can have large consequences for dryland carbon and water cycling (Li et al. 2023). Furthermore, dryland GPP and ecosystem respiration can be co‐limited by nutrients like nitrogen (Reed et al. 2012). Finally, dryland ecosystem functioning is also widely variable as driven by strong plant intra‐species and inter‐species differences especially during drought (Smith et al. 2024).

It can be challenging to estimate dryland net ecosystem exchange of carbon (NEE), and thus the balance between GPP, total ecosystem respiration (heterotrophic and autotrophic), and carbon losses through disturbances such as fire, grazing, and erosion. This includes challenges in understanding and modeling the integration of a mosaic of lifeforms across heterogeneous dryland landscapes (Table 1), including drought‐avoidant grasses, drought‐resistant woody shrubs and trees, biocrusts, and night‐functioning succulents; this includes intra‐species differences in functioning. In seeking to understand how dryland GPP, NEE, and ET dynamics respond to climate and, in turn, contribute to global carbon and water cycling, ARID aims to address: across different timescales, what are the dominant dryland mechanisms driving dryland function such as plant hydraulics, leaf‐level photosynthesis, respiration, and nutrient cycling?

#### 3.2.4 Dryland Geology and Soils

Dryland soils, sediments, and bare rock affect the Earth system in many ways and connect to the well‐being of humans, wildlife, and natural and agricultural ecosystems. For example, major global dust sources originate from arid regions and drive large uncertainties in Earth system models (Mahowald et al. 2005). Climate and human activities can create dust storms, accelerate erosion, and alter the energy balance, which in turn impact human systems and climate. Drylands also contain diverse communities of photosynthetic soils, called biological soil crusts, that cover about 12% of land surfaces and play critical roles in soil stabilization, fertility, water cycling, and carbon exchange with the atmosphere (Elbert et al. 2012; Weber et al. 2022). Finally, soil vapor adsorption and soil vapor transport play a large role in dryland evapotranspiration and moisture sources (Paulus et al. 2024).

Drylands provide opportunities to improve our quantitative and predictive understanding of soil structure, function, and responsiveness to change within drylands and beyond. Unlike densely vegetated regions of many humid locations, drylands often have vast bare soil coverage visible from air and space that can strongly affect the signal‐to‐noise of remotely‐sensed vegetation indices (Huete 1988). This can create errors for detecting vegetation‐only signals, which must be mitigated to evaluate vegetation structure and function. Conversely, widespread exposed soil patches allow development of remotely sensed soil erosion indices as well as improved mapping of soil carbon, soil texture, and critical minerals such as lithium needed for energy storage (Washington‐Allen et al. 2008). ARID aims to address: how do geology, soil physicochemical characteristics, and biological activity drive and respond to climate variability, land use, and ecosystem change?

### 3.3 Science Theme: Carbon Cycle Interannual Variability and Long‐Term Trends

Currently, about half of anthropogenic carbon emissions are removed from the atmosphere annually by natural carbon sinks, with roughly equal amounts taken up by the land and the ocean. Despite being characterized as having low organic carbon stocks on a per‐area basis, drylands contribute substantially to this carbon budget, especially with drylands' 40% of global land area coverage (Ahlström et al. 2015). Specifically, dryland contribution to global net carbon uptake is estimated to be about 20% with TRENDY models and about 40% with remote sensing MODIS‐based estimates (Ahlström et al. 2015; Sitch et al. 2024; Wang et al. 2022). Recent analysis of very high‐resolution satellite images also suggests much greater tree carbon biomass storage in drylands than previously thought (Tucker et al. 2023). Furthermore, field‐based flux towers show that most, but not all, dryland locations act as net carbon sinks on average, but oscillate between source and sink, with interannual variability of carbon fluxes in drylands often substantially larger than the mean (Biederman et al. 2017). In fact, whether drylands act as a sink or a source of carbon in any given year can strongly influence the temporal variability in the global carbon cycle and CO_2_ growth rate (Ahlström et al. 2015; Poulter et al. 2014). As such, it is understood that drylands dominate the variability of the global carbon cycle (Poulter et al. 2014). Consequently, detecting global reductions in carbon emissions requires accurately quantifying the strong year‐to‐year variability of carbon concentrations driven by drylands.

Nevertheless, dryland carbon flux estimates vary widely, are disputed, and are challenging to model and predict. Global‐scale, process‐based land surface and dynamic vegetation models often show substantial deviation from flux tower‐based net and gross carbon uptake mean and interannual variability, with considerable spread across model estimates (Fawcett et al. 2022; MacBean et al. 2021; Teckentrup et al. 2021; Wang et al. 2022). Remote sensing‐based GPP and NEE estimates perform similarly poorly in drylands (Biederman et al. 2017; Smith et al. 2019). The combined effects of short‐term pulse events, seasonal to interannual alterations of precipitation patterns, and changes in ecosystem structure add to the complexity of the dryland carbon cycle and fluxes and the representation of that complexity in process‐based models. Dryland soils also store vast amounts of carbon in organic and inorganic forms, though estimates remain highly uncertain (Lal 2004).

Addressing these issues is vital for near‐term management and policy decisions aimed at climate‐based mitigation. This ARID Science Theme aims to better understand and quantify dryland carbon cycling as well as determine the underlying processes driving this cycling. Specifically, improving estimates of the dryland contribution to the carbon cycle requires mechanistic findings (including from the other themes) to diagnose why remote sensing and model‐based carbon flux products differ in their interannual variability and trends from in situ carbon fluxes (Table 1). For example, improving the estimation of carbon fluxes and storages (and their variability and trends) requires cross‐scale estimates of key dryland processes, including, but not limited to, flux sensitivity to soil water limitation and vapor pressure deficit, ecosystem responses to rainfall pulses, soil moisture threshold related behavior, and differences in behavior within and across dominant dryland plant functional types (De Kauwe et al. 2015; Trugman et al. 2018). Observing these processes and others across the spatiotemporal scales needed for heterogeneous drylands is enabled by airborne and satellite remote sensing, which ultimately provides a means to benchmark and further develop process representation in terrestrial biosphere models and carbon flux estimates from remote sensing‐based products. The overall question this theme aims to address is: What is the contribution of drylands to the mean, trend, and interannual variability of global terrestrial carbon uptake and how large are the carbon stocks and fluxes in drylands?

### 3.4 Science Theme: Social‐Ecological Systems

Dryland systems serve as critical water and land resources and habitat for wildlife and livestock, crops, energy production, and other vital ecosystem services supporting rural livelihoods (Briske et al. 2023; McNeeley et al. 2017). Therefore, dryland systems support livelihoods associated with ranching, farming, forestry, conservation, recreation, cultural amenities, and renewable energy production; and are reliant on key ecosystem processes underlying habitat integrity, biological productivity, water resource quality and quantity, biodiversity, and soil health (Briske et al. 2023; Fernández‐Giménez et al. 2019). Dryland livelihoods and agricultural practices operate as tightly integrated social ecology systems (SES) (Havstad et al. 2007; Mccollum et al. 2017).

Variations and changes in critical ecosystem services act to constrain the ability of people to meet livelihood needs in these dryland regions. Issues with these changes are shared across U.S. public entities (e.g., Bureau of Land Management (BLM), U.S. Forest Service (USFS), etc.), Tribal land managers, and private land managers, as well as with international entities dealing with land degradation (e.g., United Nations Convention to Combat Desertification (UNCCD), Food and Agriculture Organization (FAO)). Land use practices in drylands have also altered ecosystems and have affected water availability, further exacerbating stresses to various livelihoods, such as rangeland management, cropping systems, and tourism (Briske et al. 2023).

This ARID Science Theme explores the consequences of changes in drylands for social‐ecological systems and the management strategies and options that can maintain the critical services provided by drylands even in the face of change. The overall question this theme aims to address is: what are the consequences of changes in drylands for social‐ecological systems, and what management solutions can maintain the critical services provided by drylands even in the face of change?

#### 3.4.1 Land Management

Land‐use management in drylands sustains ecosystems and their services and can safeguard the future of rural livelihoods, agricultural economics, and communities that depend on drylands. Managed lands in drylands include rangelands as well as rainfed and irrigated croplands. Notably, rangelands constitute the largest land use in the world and are predominantly located in drylands (Maestre et al. 2022). Drylands also maintain both traditional and alternative energy production, mining, recreation, and forest management, including old‐growth forests. Drylands are also managed by a range of decision makers across public and private sectors. For example, in the U.S., drylands are managed by BLM (1 million km^2^), USFS (781,000 km^2^), Tribal authorities, and private landowners. Decision makers across these entities can benefit from spatial data on their natural resources for informed decision making.

Land management of dryland systems is faced with balancing multiple objectives such as maintaining cultural assets, biodiversity, land productivity, access to water, and soil health. However, land managers are faced with interacting challenges like reduced water availability, higher temperatures, increasing wildfire, and exotic plant invasions (Table 1). This threatens the ability to manage wildlife, vegetation, and cultural resources. These challenges, combined with a lack of observations, ultimately limit options for supporting land management decision‐making. Example challenges include groundwater depletion and stream flow reductions, limiting irrigated cropland systems worldwide. New deployments of energy projects, including solar and wind power installations, compete with rangeland activities and conservation efforts and can increase water demand (Briske et al. 2023). Additionally, Tribal Nations in U.S. drylands, are facing challenges with their combined ecological interests, such as restoring buffalo populations and estimating wild horse and burro numbers throughout the western U.S. drylands. These Tribal interests and goals are often shared by other sectors. Improved ecological forecasting and “now‐casting” of dryland conditions can greatly augment practitioners' ability to deal with emerging stresses to ecosystem services. ARID aims to address: how are land and water resources as well as resource management being affected by drought and aridity changes?

#### 3.4.2 Adaptation and Mitigation Strategies

As dryland systems continue to be affected by climate and land‐use changes, the need to consider adaptation and mitigation strategies for these regions is crucial and growing. Development of climate resilient management strategies that assess multiple stresses, such as droughts, extreme heat, erosional flooding, dust storms and fires, is needed to enhance coping responses, to develop place‐based adaptation and mitigation strategies, and to enable transformative actions when needed. These climate resilient strategies need to incorporate a social ecological systems perspective that links ecosystem services to livelihood strategies as well as use co‐design and co‐production practices that outline potential options and actions. We outline adaptation and mitigation strategies separately.

##### 3.4.2.1 Adaptation

As changing conditions in dryland regions affect the delivery and availability of key ecosystem services, such as forage, browse, fuel wood, grains, habitat, and water resources, the use of adaptation strategies is becoming more urgent. For example, social‐ecological systems research studies have incorporated improved drought assessments to enhance drought response strategies co‐developed with natural resource managers (Fernández‐Giménez et al. 2019; McNeeley et al. 2017). These analyses provide greater insight into the range of coping and adaptation choices to make under various levels of adaptive capacity. They also highlight the importance of co‐development practices to be used for greater end‐user usage of research. Further efforts are required to enhance the resilience of communities to adapt to flash drought, increased fire events, and loss of land productivity. This requires engaging with operators at the local scale to enhance the delivery of more nuanced information needed to deal with these climate stresses, given local adaptive capacity. ARID aims to address: how can dryland ecosystems enhance their resilience in the face of environmental stressors, and what adaptive strategies can local communities implement to build resilience?

##### 3.4.2.2 Mitigation

Dryland systems are being evaluated for nature‐based solutions to enhance carbon sequestration and establishing renewable energy sites, such as solar and wind. These land use practices, when integrated into livelihood goals and strategies, can enhance the socio‐economic resilience of many communities. Considerations of impacts on wildlife habitat, water availability, and further fragmentation of landscapes should be addressed in the permitting and siting of large‐scale energy facilities. Various natural resource management approaches need further development to take advantage of these potentials and to contribute to various livelihood systems while not undermining cultural norms and needs. ARID aims to address: how will climate variability and water limitations impact carbon sequestration efforts?

## 4 Multi‐Scale, Multi‐Method Approach

**FIGURE 3:**
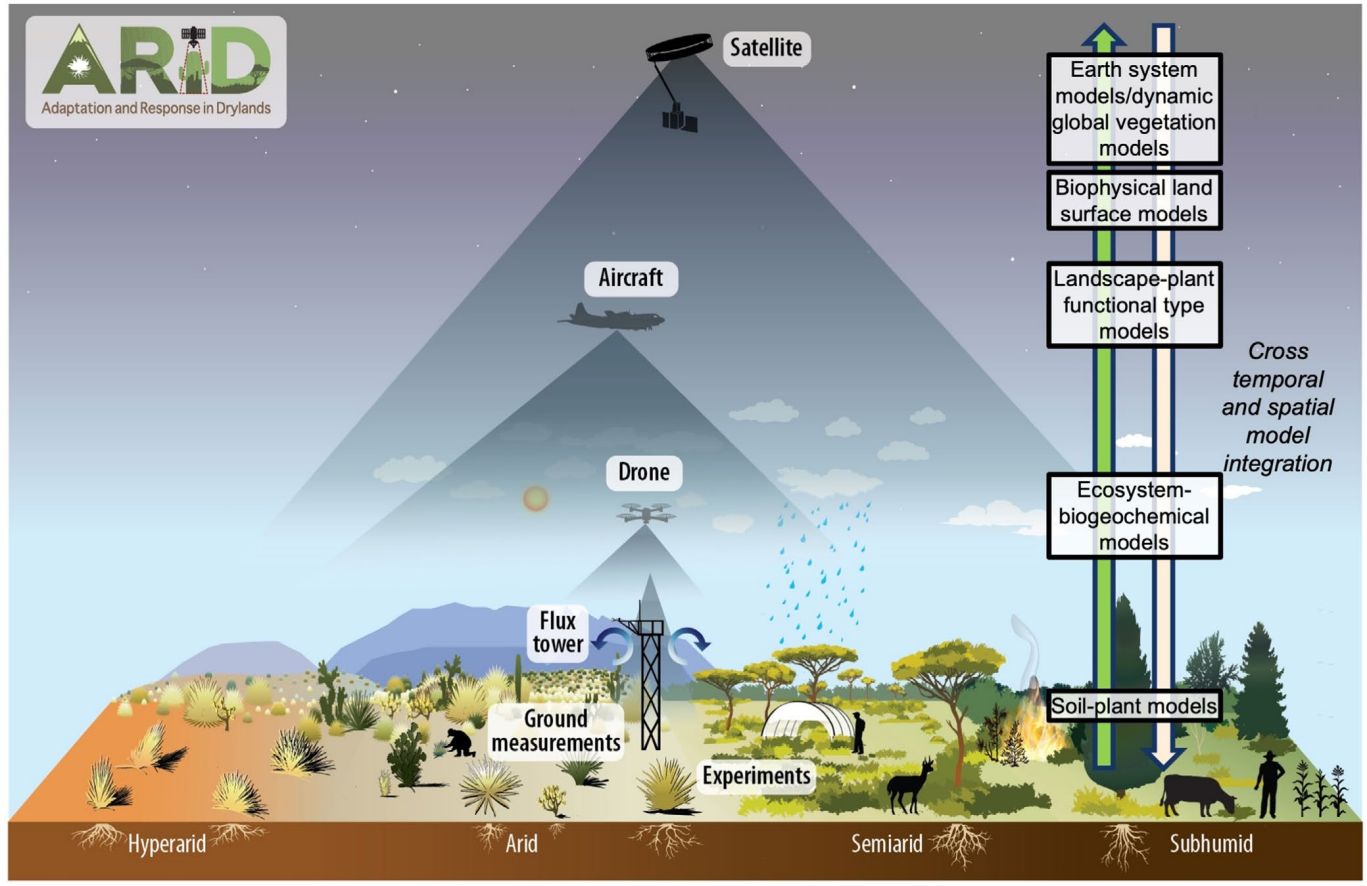
ARID's multi‐scale approach to observe dryland ecosystems' high spatiotemporal heterogeneity with new sub‐orbital observations that bridge ground and satellite measurements. The measurements can be informed by and integrate within a range of models. The vertical placement of the modeling approaches approximately corresponds to the scale of the measurements. This figure is adapted from figure 1 in Feldman, Feng, et al. (2024), Feldman, Reed, et al. (2024) (DOI: https://doi.org/10.1029/2024EF004811) with permission from AGU Earth's Future, Copyright 2024 American Geophysical Union. This figure is reproduced strictly for academic purposes.

In this section, we discuss approaches and guiding principles to address challenges with remote sensing, modeling, and ground observations in drylands. We identified the observational challenges and gaps, how sub‐orbital measurements can help fill those gaps, and where sensor and scale fusion is needed. The approach includes measurements across spatial, temporal, and spectral axes to observe drylands' highly spatiotemporally heterogeneous landscapes as well as integrate within process modeling (Figure 3). We emphasize that these approaches are multi‐scale, use multi‐sensor integration, and can be applied across domains and focal areas.

### 4.1 Remote Sensing Strategy

Drylands have consistently played impactful roles in remote sensing development. Due to less cloud cover and higher likelihoods of favorable atmospheric conditions, many remote sensing techniques were developed in drylands, including early retrievals of surface reflectance and estimates of vegetation condition using red and near‐infrared bands, which would later become the Normalized Difference Vegetation Index (NDVI) (Tucker 1979). Despite this important role, our remote sensing capacity for core measurements of ecosystem structure, composition, and function (e.g., productivity, water exchange, etc.) in drylands show substantial deficiencies (Biederman et al. 2017; Smith et al. 2019).

Drylands are characterized by high spatial heterogeneity and high temporal variability which pose unique remote sensing challenges, including several we list here. (i) The characterization of dryland composition and function is difficult without very high‐resolution imaging spectrometer data due to dynamic mixtures of plant lifeforms (grasses, shrubs, forbs, trees), mineral soil, and non‐vascular biocrusts. (ii) Traditional remote sensing‐based ET and GPP products rely on fixed biome‐level parameters such as maximum light use efficiency which can vary greatly across dryland function types and introduce substantial errors in GPP estimates. (iii) Traditional remote sensing‐based ET and GPP products rely heavily on optical reflectance‐based estimates of greenness such as fraction of absorbed photosynthetically active radiation (FAPAR), which captures changes in photosynthetic potential rather than photosynthetic activity or efficiency. This leads to additional errors in GPP estimation. (iv) Historic remote sensing platforms do not provide high enough temporal resolution to fully capture episodic dryland vegetation activity due to irregular rainfall, limiting our ability to resolve temporal variability in ecosystem structure (e.g., plant area index) and function (e.g., GPP, ET). (v) Soil moisture estimates, often from low resolution satellites and model reanalysis (model‐observation integration), do not capture high spatial–temporal variability in drylands required to model and monitor water fluxes.

To address these challenges, the ARID dryland campaign proposes a multi‐scale, multi‐platform, and multi‐sensor framework (Smith et al. 2019), including co‐use of site‐level proximal instruments, UAS, aircraft flights, and existing satellites. The multi‐scale ARID campaign will enable the development of improved algorithms and models for estimating essential physical parameters and reliable higher‐level products (e.g., GPP, ET) that can be applied globally. As outlined below, we now have the multi‐scale instrumentation and technology to revolutionize our understanding of drylands and the services they provide, and a core aim of the ARID campaign is to provide the infrastructure and coordination to enable such a revolution. A key deliverable of this multiscale effort will be targeted information on spectral, spatial, temporal, radiometric resolution needs that could inform more ideal future satellite missions that target better dryland monitoring from space.

#### 4.1.1 Physical Parameter Priorities

The ARID scoping team defined a list of physical parameters that are required to address science questions within each science theme and sub‐theme (Table S2). These include variables like soil moisture, ET, surface temperature, vegetation height, plant functional type coverage, aboveground biomass, vegetation water content, invasive species cover, solar‐induced fluorescence, and GPP. Successfully observing these parameters during the ARID campaign at the appropriate spatio‐temporal resolutions would contribute to substantial advancement of dryland science across many focal areas and better interpretation of existing satellite measurements. As detailed below, the proximal, UAS‐based, and airborne remote sensing approaches can be partly focused on scaling up and validation of variables that might have existing retrieval or estimation approaches, but are not currently sensed with sufficient spatial or temporal resolution by satellites (Pierrat et al. 2025; Schimel et al. 2019). For example, the inherent spatial averaging that occurs through space‐based observations often cannot resolve the complex diversity of plant functional types and vegetation structure. Furthermore, some parameters listed in Table S2 such as soil carbon and belowground biomass remain a significant challenge to estimate from direct observations. Others are outputs from more uncertain, multi‐step retrieval processes with more built‐in assumptions, such as for remote sensing‐model based GPP and ET estimates that require several input parameters (such as chlorophyll fluorescence, surface temperature, light use efficiency, precipitation, soil moisture, and PFT fractional cover) (Table S2). The approaches below are designed to address these different types of gaps.

#### 4.1.2 Proximal Remote Sensing

Proximal remote sensing uses sensors, analogous to those on aircraft and satellites, that are deployed in situ to provide high spatial, temporal, and spectral resolution measurements enabling potentially the most direct link to dryland field measurements including flux measurements from eddy‐covariance towers (Pierrat et al. 2025; Schimel et al. 2019). Since they use the same spectral measurements as that of aircraft and satellites, proximal sensors can be used to develop relationships between remotely sensed information and key parameters mentioned above while validating satellite measurements and products (Zhang et al. 2023). For example, retrieval methods for surface features like biocrust, plant biodiversity, canopy level function, and soil nutrients from proximal measurements of reflectance spectra can be developed via enhancing physical models and/or training machine learning models (Ji et al. 2026). Such steps can be later used to scale up these properties using aircraft or remote sensing measurements. As a part of the ARID field campaign, we propose the use of five different types of proximal sensing for observing ecosystem structure, composition, and function: visible‐to‐shortwave infrared spectral reflectance (VSWIR), solar‐induced chlorophyll fluorescence (SIF), thermal infrared emittance (TIR), microwave backscatter (WVB), and light detection and ranging (lidar).

#### 4.1.3 Uncrewed Aircraft Systems (UAS) Remote Sensing

While covering smaller spatial extents than aircraft and satellites, UAS‐based (i.e., drone) assessments of ecosystems allow finer spatial scale and more frequent data acquisitions (Sun et al. 2021). They have shown utility for remote sensing algorithmic development, particularly when paired with coordinated field campaigns (Chadwick et al. 2020). UAS platforms and sensors include VSWIR, SIF, TIR, and WVB and are evolving very rapidly. With their hyperspatial resolution (5 cm to 1 m) and on‐demand, rapid data acquisition, UAS can play a key role in the ARID field campaign in capturing the high spatial heterogeneity at flexible intervals to investigate plant function during rapid green‐up, senescence, or pulse events. UAS thus provides the on‐demand flexibility to capture frequent data at priority field and flux tower sites especially during abrupt events (rain pulse, heat wave). They can be used to scale up field and proximal remote sensing measurements to airborne (1–15 m) and space‐based measurements (> 10 m). For example, high resolution fractional plant cover of different plant types can be generated from the fusion of drone measurements with image spectroscopy and lidar data from satellites (Sankey et al. 2018), which can used to develop mixture models that accurately estimate these fractional covers (Pervin et al. 2022).

#### 4.1.4 Airborne Remote Sensing

Airborne sensors fill the intermediate spatial scale between field or UAS data and space‐based sensors. While airborne data have been acquired in parts of western U.S. drylands, ARID scoping showed that these efforts seldom use the data to advance dryland science. Additionally, previous NASA airborne campaigns typically only acquired one overpass per area, while ARID scoping revealed that dryland science questions require multi‐temporal, seasonal, and inter‐annual repeat acquisitions to capture the high temporal variability of drylands. To accomplish a multi‐temporal approach, ARID can utilize a combination of foundational NASA aircraft‐based lidar, thermal, hyperspectral, and microwave sensors and partner sensors (e.g., NEON‐AOP), all of which are at the resolutions needed to address dryland science questions (Table 1). Namely, multi‐date airborne measurements could be achieved by coordinating with current campaigns and ongoing investments. This includes complementing, for example, National Ecological Observatory Network (NEON) Airborne Observation Platform (AOP) peak‐greenness measurements with 2–3 additional measurements in shoulder seasons. Another strategy is acquiring weekly drone‐based imaging spectrometer data over field sites with a time series of measurements (e.g., flux towers, long term measurement networks) within footprints of aircraft overflights. Consequently, time series of spatial airborne overpasses can be estimated with the temporal information from time series of ground measurements and/or frequent drone fly‐overs within the footprint using AI‐based and other spatial methods. Such artificial intelligence methods can also be used to spatially downscale single aircraft overpasses with the higher resolution drone measurements (Ji et al. 2026).

#### 4.1.5 Satellite Remote Sensing

The current (and future) space‐based sensors can retrieve parameters near or at the sufficient spectral, spatial, and temporal resolutions across vast global drylands to address the ARID science questions and themes (Table 1). They also measure VSWIR, SIF, TIR, and WVB that are available from proximal, UAS, and aircraft sensors and needed to measure key variables to address the ARID science questions (Table S2). The ARID field and airborne campaign can enhance retrieval algorithm development of key parameters from satellites, for example, by providing in situ and airborne measurements needed for satellite‐based calibration and validation. Given the high spatiotemporal complexity of dryland landscapes, a better understanding of drylands globally will require a full interpretation of current and future spaceborne sensors and their increasingly higher‐resolution capabilities spectrally, spatially, and temporally. We discuss several approaches for addressing dryland science questions using the available spaceborne sensors. While we focus primarily on NASA sensors, a range of spaceborne instruments is becoming available across the world's public and private sectors that can address these dryland questions.

- Long‐term trends and variability of global drylands can now be viably captured with 10–40 year records of vegetation greenness and land surface temperature (i.e., Landsat), soil and vegetation moisture content (i.e., Soil Moisture Active Passive mission (SMAP)), vegetation structure (ICESat), and photosynthesis (Orbiting Carbon Observatory 2 (OCO‐2)). These platforms provide critical observations at a fixed overpass time and can be ideal for evaluating vegetation function, phenology, and long‐term trends in vegetation dynamics. An important example is evaluating the extent and magnitude of global greening of vegetation in semiarid areas (Fensholt et al. 2012).
- Satellites with frequent temporal revisits can help address the challenge of capturing the high temporal variability of dryland ecosystems through novel data fusion techniques and use of geostationary satellite platforms. As a salient data fusion technique, the NASA Harmonized Landsat and Sentinel‐2 (HLS) initiative integrates observations from several Landsat and Sentinel satellites to generate a reflectance data product at 30‐m spatial resolution and two‐to‐three‐day sampling. Geostationary satellites provide minute‐to‐hourly information at a fixed position, seamlessly capturing diurnal to interannual dynamics over a given continent. This includes coverage of global drylands in North and South America (i.e., GOES‐R, TEMPO), Central Asia and Australia (i.e., Himawari‐8/9) and Africa, the Middle East, and Southern Europe (i.e., SEVIRI). Together, these novel platforms and initiatives offer invaluable yet underexplored insight into the rapid pulse dynamics and flash drought response of dryland ecosystems (Table 1).
- Sensors on the ISS capture both high spatial and temporal variability of global drylands. They have a high capacity to evaluate dryland's heterogeneous surfaces with their mostly higher spatial resolutions (< 100 m). These instruments include ECOSTRESS (70‐m) measurements of LST and ET and OCO‐3 (2‐km) measurements of SIF. The revisits at diurnal and daily scales provide unique capabilities to evaluate leaf level functioning under diurnal cycles of radiation and temperature and post‐rainfall dry spells (Zhang et al. 2023). Three ISS‐based instruments (EMIT, DESIS, and HISUI) are providing first‐time space‐borne hyperspectral measurements that can be used to map dryland spectral traits across diverse functional groups (e.g., plants with C3, C4, and CAM photosynthesis pathways, diverse biocrust species). Finally, the GEDI mission is providing novel structural traits (e.g., vegetation height, plant area index, and total aboveground biomass) that capture the high structural biodiversity, especially of dryland forests with both open and closed canopies. These instruments offer observations at variable overpass times and thus can be jointly used to gain insights into diurnal dynamics at high spatial resolution.
- Commercial small satellite constellations provide hyperspatial and hyperspectral measurements. Emerging polar‐orbiting small satellite constellations have the potential to provide first‐time high spectral and spatial resolution observations in the optical and thermal regions of the electromagnetic spectrum. These measurements are often at the highest spatial resolutions (< 10 m) and enable more accurate vegetation mapping in sparsely vegetated dryland regions.

### 4.2 Modeling Strategy

Models are pivotal for improving our knowledge of the role of drylands in global carbon and water cycles and land‐atmosphere feedbacks. They are also tools for forecasting the effects of weather extremes and climate and land use changes on dryland ecosystems worldwide. However, our dryland ecosystem process understanding and modeling all remain poor, in part from ecological results from mainly mesic areas being historically incorporated into land surface schemes. Thus, there is an urgent need to assess where and why models are not performing well, in part, by comparing and constraining models across scales with field, airborne, and satellite data from dryland field campaigns. Ultimately, multiscale observational platforms and measurement campaigns envisaged in ARID are the key to bridging ecosystem theory and models to address ARID science themes (Table 1).

During the ARID campaign, multi‐scale remote sensing and ground observations can be used to enhance dryland representation of mechanisms in models at a variety of scales from local sites to global Earth systems (Figure 3). Dryland modeling can also be used to inform dryland measurement collection. Such dryland modeling enhancement activities are expected to improve our ability to test research hypotheses and address long‐standing dryland scientific questions (Table 1), as well as enhance decision frameworks, especially for rangeland management in the western U.S. (Watts et al. 2025).

#### 4.2.1 Key Dryland Modeling Limitations

Currently, most parameterizations and parameters in global scale land models have been calibrated for more mesic ecosystems and likely have limited representation of dryland ecosystem processes and dryland plant and soil traits. Therefore, a considerable number of processes (some highlighted below) needed to accurately represent dryland ecosystem structure, functioning, and dynamics are not necessarily included or could be misrepresented. Furthermore, knowing which processes are important is a considerable challenge. Enhanced representation of surface characteristics and understanding of complex dryland processes gained in a field campaign can be used to identify and address several modeling limitations (Tables 1 and S2).

For example, representation of the spatial complexity of mixed woody‐herbaceous ecosystems and associated processes (e.g., shrub‐grass competition for water) is critical for drylands but is largely absent from global modeling frameworks. The methods used to create global scale PFT maps used to prescribe PFT type and fractional cover in global land models have the highest uncertainties in heterogeneous, sparsely vegetated dryland regions, which results in a large spread in modeled carbon, water, and energy fluxes due to uncertainties in PFT fractional cover (Hartley et al. 2017). Furthermore, models that predict dynamically changing PFT type and cover over time appear to overestimate dryland carbon flux variability and would benefit from careful benchmarking against independent PFT fractional cover maps (Pervin et al. 2025).

Other key processes are either only recently under model development and have not yet been widely tested for drylands, or are missing in models altogether. Their specific contribution to dryland model uncertainty is less known. These include dryland ecosystem responses to rainfall pulses, water stress, carbon flux variability, woody plant encroachment, and vegetation recovery from disturbance (e.g., fires) and climate extremes (e.g., severe drought) (Baudena et al. 2015; Whitley et al. 2017). For example, most global land models use an empirical water stress function, important for representing water‐limitation in drylands, that performs poorly in capturing plant water stress (De Kauwe et al. 2015). This water stress representation diverges greatly across models (Rogers et al. 2017), adding potentially substantial uncertainty for dryland water stress representation. Furthermore, with regard to pulse dynamics, complex soil microbial and photosynthetic upregulation (Birch effect, pulse‐reserve) can occur after episodic dormancy (Ogle and Reynolds 2004). Unique dryland model development may be required to capture these pulse processes that are governed by the timing, magnitude, and antecedent conditions of rainfall events rather than seasonal climate variations.

#### 4.2.2 Strategy for Improving Dryland Model Process Understanding and Outputs

ARID's modeling strategy entails (a) diagnosing and improving model representation of physical processes, (b) hypothesis testing with models of different scale and capability, (c) observation‐model data fusion approaches that improve state and flux estimates, and (d) use of observing system simulation experiments (OSSEs) to inform data collection needs. These strategies inform one another to varying degrees. While these strategies can be viewed as general for any modeling effort during a field campaign, the dryland modeling strategies here prioritize the use of in situ and high‐resolution aircraft and satellite measurements for the diagnosis of model processes and parameterizations.

##### 4.2.2.1 Model Process Representation Diagnosis

A dryland field campaign would enable cross‐scale measurements for resolving and benchmarking dryland‐specific processes anticipated to be misrepresented by or missing entirely in global land models and poorly constrained in ecosystem and landscape scale models (Tables 1 and S2). For example, flux tower and satellite‐based observations offer an opportunity to benchmark model‐based relationships between fluxes and soil moisture across heterogeneous landscapes, which is a critical uncertainty in Earth system modeling (Trugman et al. 2018). Additionally, pulse chasing measurement campaigns can be used to coordinate in situ and aircraft measurements during and after rain events (Table 1) to evaluate lagged pulse responses to rainfall that models may not be able to capture. Other examples to be targeted for model benchmarking with campaign measurements include complex interactions between fire and dynamic vegetation in mixed shrub‐grass ecosystems, the role of biocrust composition and structure in sparsely vegetated, heterogeneous ecosystems, dryland plant‐specific phenologies related to winter and/or summer desert rain seasons, and dynamic root water uptake influenced by seasonality, extreme weather, and competition.

##### 4.2.2.2 Cross‐Scale Modeling

Given highly spatially heterogeneous landscapes and uncertain representation of dryland processes, use of models with varying spatial scales and varying complexity is necessary within the ARID campaign. This approach focuses on diagnosing and enhancing model process shortcomings at the anticipated scales of representation, rather than developing a single modeling framework. Simpler or reduced‐complexity models can be used to isolate and test competing hypotheses about dryland process controls at the stand to landscape scale, aided and benchmarked with field or high resolution airborne and satellite observations. More comprehensive land‐surface and Earth system models can assess the broader implications of those mechanisms for carbon, water, and energy exchange, and can be benchmarked with coarser resolution satellite data products. By spanning plot, landscape, and regional domains, ARID enables multiple modeling approaches to be applied to the same physical and ecological processes rather than relying on a single scale or framework, and can inform cross system interactions (Figure 3).

##### 4.2.2.3 Model Data Fusion

By fusing process models with remote sensing data, models can be constrained and adjusted, improving the accuracy of predictions related to above‐ and belowground ecosystem processes. For example, former NASA field campaign (ABoVE) estimates of above ground biomass or leaf area derived from remote sensing were used to reduce bias in model biomass and improve estimates of regional carbon flux and belowground carbon pools (Huo et al. 2024). This approach not only improves model performance but also allows for better understanding of complex ecosystem dynamics and which processes are missing or misrepresented in models (e.g., Mahmud et al. 2021), particularly in heterogeneous environments where direct measurements are limited. Additionally, integrating in situ observations with model predictions enables estimating quantities that cannot be fully or confidently observed remotely, including belowground soil and root processes and carbon fluxes (Table 1). Other model‐data fusion techniques, including artificial intelligence approaches and data assimilation, can also be used to enhance the utility of remote sensing data for developing estimates of soil moisture, temperature, vegetation condition, vegetation structural attributes, eco‐physiological processes, biodiversity characterization, and others across various dryland ecosystem types (Table S2).

##### 4.2.2.4 Observing System Simulation Experiments

The use of models in Observing System Simulation Experiments (OSSEs) can also play a crucial role in planning for data collection by providing a quantitative means to optimally select field and aircraft sampling locations. OSSEs are also useful in creating simulation environments that can evaluate the precision and resolution of emerging remote sensing technologies, especially by providing uncertainty estimates. Finally, they can aid in the development of algorithms for retrieving land surface parameters and planning future spaceborne missions.

#### 4.2.3 Modeling Implementation Strategy

At the recommendation of members of former modeling efforts during NASA and other field campaigns, ARID proposes a coordinated and continuous modeling effort throughout the field campaign, especially via a core ARID model team that coordinates and connects community modeling activities. It is the responsibility of both the core ARID modeling team and individual research teams to coordinate efforts to share insights and synthesize efforts.

The core ARID model team will oversee coordination of model evaluation and development in three phases. The first phase focuses on diagnosing model process shortcomings and developing linkages between models and observations (e.g., ensuring observation operators are adequate to match model processes with observed quantities). Dryland‐specific process representations in models, especially those expected to be misrepresented, can be tested across different model scales and complexities by comparing field campaign observations to model outputs and states as well as emergent relationships. More specific, finer scale models can be assessed first, and those findings used to test larger scale models. Additionally, uncertainties can be quantified through integrating field campaign data with models, especially at the fine scales that can capture the relevant processes, to update model states, constrain parameters, and/or to rigorously benchmark model‐based relationships. Diagnostic and data integration efforts in parallel are expected to identify priorities for further model development and model‐data integration efforts.

The second stage includes adding relevant dryland processes or altering key model parameterizations based on shortcomings identified in the first stage. Given the sheer number of processes we expect need to be modified or added to achieve accurate predictions of dryland ecosystem processes, model developments initially would be carried out factorially within one model to rigorously test the benefit of adding or modifying each process and how it impacts other processes within the model. However, such developments should be made with full cooperation and coordination with the core ARID model team and other participating model groups. Code for model developments tested carefully within one model would be expected to be shared widely with all model groups. Model developments carried out in isolation within different modeling groups cannot provide the advances in dryland modeling we need to more reliably answer pressing dryland science questions. After sufficient testing of key processes, these processes can be tested and implemented on models across scales to prepare for the third stage.

Finally, in the third stage, the new state‐of‐the‐art dryland modeling schemes that were further developed and optimized using data collected throughout the dryland field campaign can be used to address hypotheses across ARID science themes (Table 1). For example, they can be used to make more reliable predictions of the impact that environmental variations and trends can have on dryland ecosystems from near term to decadal and centennial time scales, and from ecosystem to global spatial scales. These predictions can be compared to those without the influence of updates made based on field campaign measurements to further understand the effects of dryland model processes on output states and fluxes and their emergent relationships.

### 4.3 Field Measurement Strategy

In situ observations of surface states and fluxes aligned with airborne campaigns and satellite observations are essential for addressing ARID scientific questions. One of ARID's strengths is the ability to leverage rich networks of available ground measurements, especially in the western U.S., of soil moisture, water fluxes, carbon fluxes, and vegetation states. These variables are all targets of remote sensing platforms. Observational and manipulative experimental data offer direct means to address long‐standing knowledge gaps for dryland ecosystems at representative sites, and for improving interpretation and use of remote sensing data. A goal for ARID is to systematically combine ground measurements with proximal, aerial, and satellite remote sensing as well as modeling efforts to quantify dryland functioning and forecast future responses across dryland regions.

Unique challenges in drylands include the ability to monitor key parameters and processes to understand mechanisms across vast landscapes that have large spatial complexity and heterogeneity. ARID can leverage the richness of existing infrastructure, including weather stations, flux towers, manipulative experiments, and other in situ networks, as well as a wealth of existing datasets including soil assessments and long‐term vegetation monitoring. However, critical new components proposed here can facilitate effective coordination of ground measurements with modeling and remote sensing efforts. We discuss these strategies here. Across these strategies, measurement priorities include sap flux, tree growth, soil moisture profiles to a depth of 1 m, belowground carbon, rooting depth, plant water status, and direct measures of net primary productivity (Table S2).

#### 4.3.1 Vision for Effective Upscaling Strategies and Improving Sensing Capabilities

ARID's ground‐based measurements are ultimately the highest resolution and thus most essential for upscaling high dryland heterogeneity of structure and functioning across the globe as well as testing our remote sensing capabilities. Using the ground measurements in our domains, ARID will use several strategies for upscaling process understanding and sensing techniques. (i) UAS and aircraft fly‐overs of in situ networks, experiments, and particularly ARID super sites present unique opportunities to calibrate and validate retrieval methods of target physical parameters using the in situ knowledge, fuse the field‐based data with remote sensing measurements to create data products, as well as understand the spatial scales of representation of the target physical parameters. (ii) The ground measurements leveraged in ARID present a unique opportunity to test sensing capabilities and innovative development of proxies for remotely sensing variables that are challenging to observe. These variables include evapotranspiration, carbon uptake of both soil and vegetation, soil carbon, soil types, deeper soil moisture, and other belowground variables. (iii) Experimental manipulation sites provide opportunities to test whether observation‐based approaches with satellites (often using statistical and artificial intelligence models) can replicate results from manipulations, such as rainfall mean and variability manipulations (e.g., DroughtNet) (Beier et al. 2012). (iv) Gaining an understanding of spatial variability and representation of target physical variables from these ground measurements (augmented with UAS and aircraft measurements) presents an opportunity to develop quantitative frameworks for optimal placement of new sensors in existing measurement networks.

#### 4.3.2 Super Sites and Distributed Flux Towers

We propose targeted augmentation of a subset of existing eddy covariance tower sites to be designated as “ARID super sites”, or sites that have many types of instruments and maintained long term measurements in a single location. Most existing flux towers in dryland sites make simultaneous ground‐based observations, including local meteorology, land‐surface water vapor, CO_2_ and energy exchanges, and soil moisture and temperature states (Baldocchi et al. 2001). ARID super sites will be selected from among long‐term flux tower networks within the ARID domain (i.e., AmeriFlux, NEON, etc.) that are more accessible, contain extensive instrumentation, and multi‐year time series across environmental gradients. These sites can also be augmented with measurements of soil moisture profiles, soil CO_2_ efflux, hyper or multispectral data, thermal data, biocrusts, and vegetation composition, cover, and structure.

#### 4.3.3 In‐Situ Networks and New Relocatable Towers

In addition to regular distributed sites and super sites, both modeling and remote sensing efforts will take advantage of a number of existing in situ measurement networks in drylands like the National Soil Moisture Network (standardized, distributed soil moisture) and Phenocam networks. Relocatable towers can be used for short‐term deployments of diverse instrument suites to test specific hypotheses. These include the ability to map eddy covariance, spectrometry, and RGB camera systems using scanning point measurements of user‐specified targets (i.e., vegetation, ground, and sky).

#### 4.3.4 Manipulative Field Experiments and Long‐Term Ecological Field Sites

We can leverage existing field‐scale manipulative experiments where key environmental variables such as precipitation amount, timing, temperature, and nutrients are manipulated to directly test and observe how and why these variables alter dryland function (Zhang, Biederman, Pierce, et al. 2021). Existing precipitation manipulations in drylands across the U.S. include RainMan (Javadian et al. 2023), DroughtNet (Smith et al. 2024), and Mean Variance Experiment at the Sevilleta (LTER). These leveraged manipulative field experiments will provide the means to test the ability of remote sensing platforms to detect and quantify known change.

#### 4.3.5 Vegetation Surveys

Within the U.S. and internationally there is a wealth of long‐term data assessing plant and biocrust community composition and cover across time and space in drylands. For example, the BLM's Assessment, Inventory, and Monitoring (AIM) program, USFS Forest Inventory and Analysis (FIA) plots, and the National Park Service's Inventory and Monitoring program have thousands of sites in the western U.S. where plant and biocrust data are collected. These existing datasets can be blended with remote sensing data in the program of record and with the collection of new data, including ground‐based, UAS, and airplane remote sensing, to improve the understanding of the controls over and changes to vegetation patterns and to build predictions for future change. This includes assessment of changes to biodiversity, increases in the abundance of exotic invasive plants, and losses or gains of functional types that lend insight into the kinds of vegetation most likely to be successful in a warmer, drier world. The sites also allow for improvement of remote sensing interpretation through validation with vegetation survey data and informing emerging new opportunities for remotely sensing (i.e., biocrusts).

#### 4.3.6 Soil Properties

Advancing our understanding of dryland controls and resilience must include consideration of the role that soils play in regulating ecosystem function and response to change. Soil maps and field‐based studies can join with remote sensing tools to provide an improved assessment of soil controls over dryland structure and function. Remote sensing of soils is challenging, and since drylands have a large coverage of soils, ARID presents a unique opportunity to both understand their role in dryland processes from rich networks of in situ measurements as well as develop and test techniques to remotely sense soil types and soil carbon. For example, although deeper soils and integrated soil carbon stocks are not currently able to be assessed with remote sensing, we can link remotely sensed to field‐based soil collections using machine learning, as well as inform emerging soil‐focused remote sensing tools.

## 5 Campaign Strategy and Guiding Principles

In order to address ARID's fundamental and applied science questions (Section 3; Table 1), we have designed an interdisciplinary, multi‐scale field campaign study that uses key dryland methodology (Section 4), which we describe here. The study domain will span a range of drylands from hyperarid to subhumid, in the U.S. and internationally. We describe our dryland domain and focal area motivation, develop our field sampling strategy within these domains using both new measurements and the existing assets, and discuss our proposed applied science activities to accomplish land management and mitigation goals.

### 5.1 Domain Selection

#### 5.1.1 Definitions and Criteria

**FIGURE 4:**
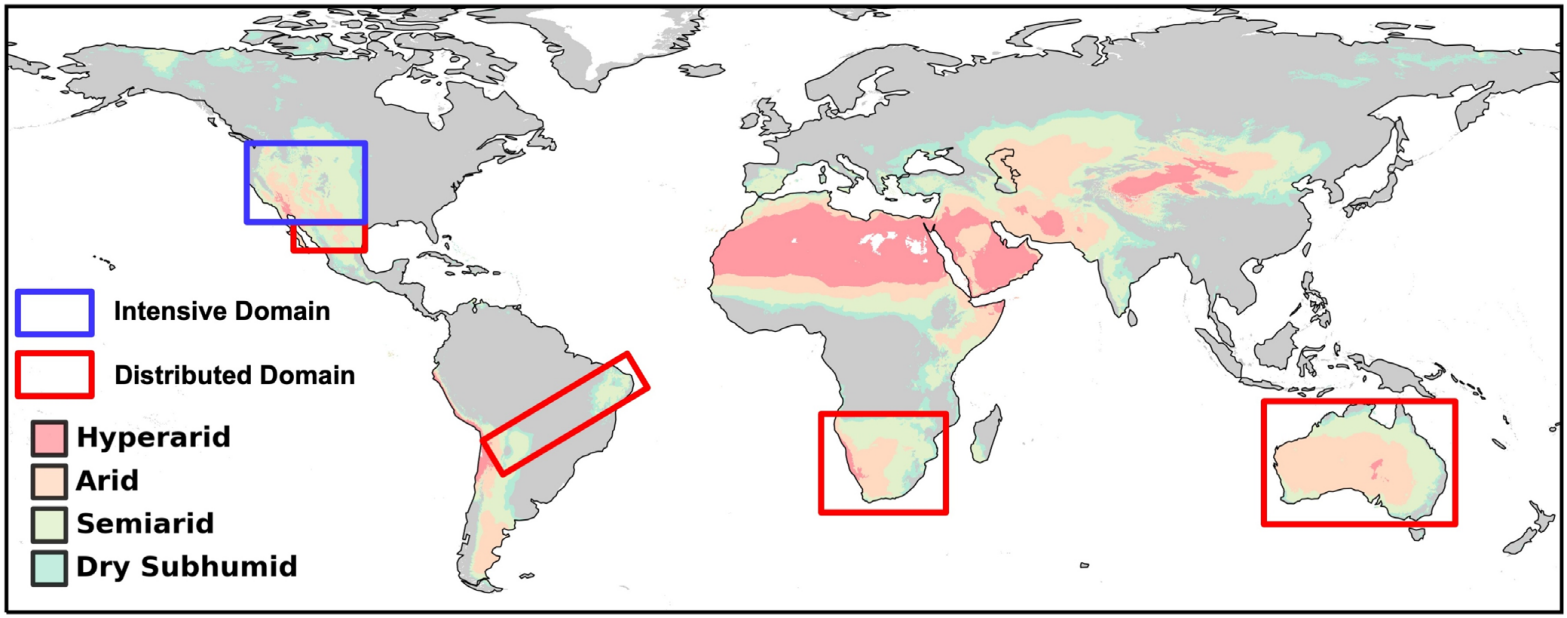
Global dryland distribution based on aridity index (AI) and each shaded region defined as hyperarid (AI < 0.05), arid (AI = 0.05–0.2), semi‐arid (AI = 0.2–0.5), and sub‐humid (AI = 0.5–0.65). Priority ARID field domains are outlined by borders and include shaded dryland regions (based on aridity index) within those borders. A core intensive domain includes shaded drylands within the western U.S. highlighted approximately by the blue borders. Candidate distributed international domains are shown as shaded regions outlined by red borders. These are ultimately areas that ARID can focus on, and are not meant to be exclusive; all shaded areas (based on aridity index) are included in ARID's domain and can be expanded (refer to text). While the campaign sampling can focus on these regions, research and measurements can still take place outside of these bounds. Map lines delineate study areas and do not necessarily depict accepted national boundaries.

Among many climatological definitions, drylands are most commonly defined as regions with an aridity index (mean annual precipitation/potential evapotranspiration) of less than 0.65 (Wang et al. 2022). Overall, ARID scoping elucidated a need to address dryland science questions (Section 3) in as wide an expanse of regions as possible, including traditionally defined drylands (Figure 4), those that are transiently dry (i.e., due to seasonality), and those that are projected to become drylands. These science questions also benefit from evaluating gradients between dryland and wetter ecosystems to gain context about the dryland dynamics and potential transitions into drylands (Grünzweig et al. 2022). As such, dryland evaluations should not be restricted to those defined based on the aridity index (Figure 4).

The goal of a remote sensing‐focused field campaign is to sample a limited region in order to gain a scalable understanding of other dryland regions. This is ultimately challenging for drylands because of their large land coverage and high diversity of vegetation species, soil conditions, topographical effects, and seasonalities. Under known challenges of optimally selecting a dryland domain, we have defined our domain criteria as regions with dryland representativeness, existing infrastructure, supporting personnel, community interest, and campaign feasibility. Based on these criteria, the ARID field campaign adopted a strategy for detailed, geographically focused and comprehensive field, airborne, and orbiting data collection in an *intensive* research region of the western US, with complementary collections at *distributed* field sites representing global drylands in northern Mexico, southern Africa, Australia, and parts of dry South America (Figure 4).

#### 5.1.2 Intensive Domain and Leveraging of Networks

**FIGURE 5:**
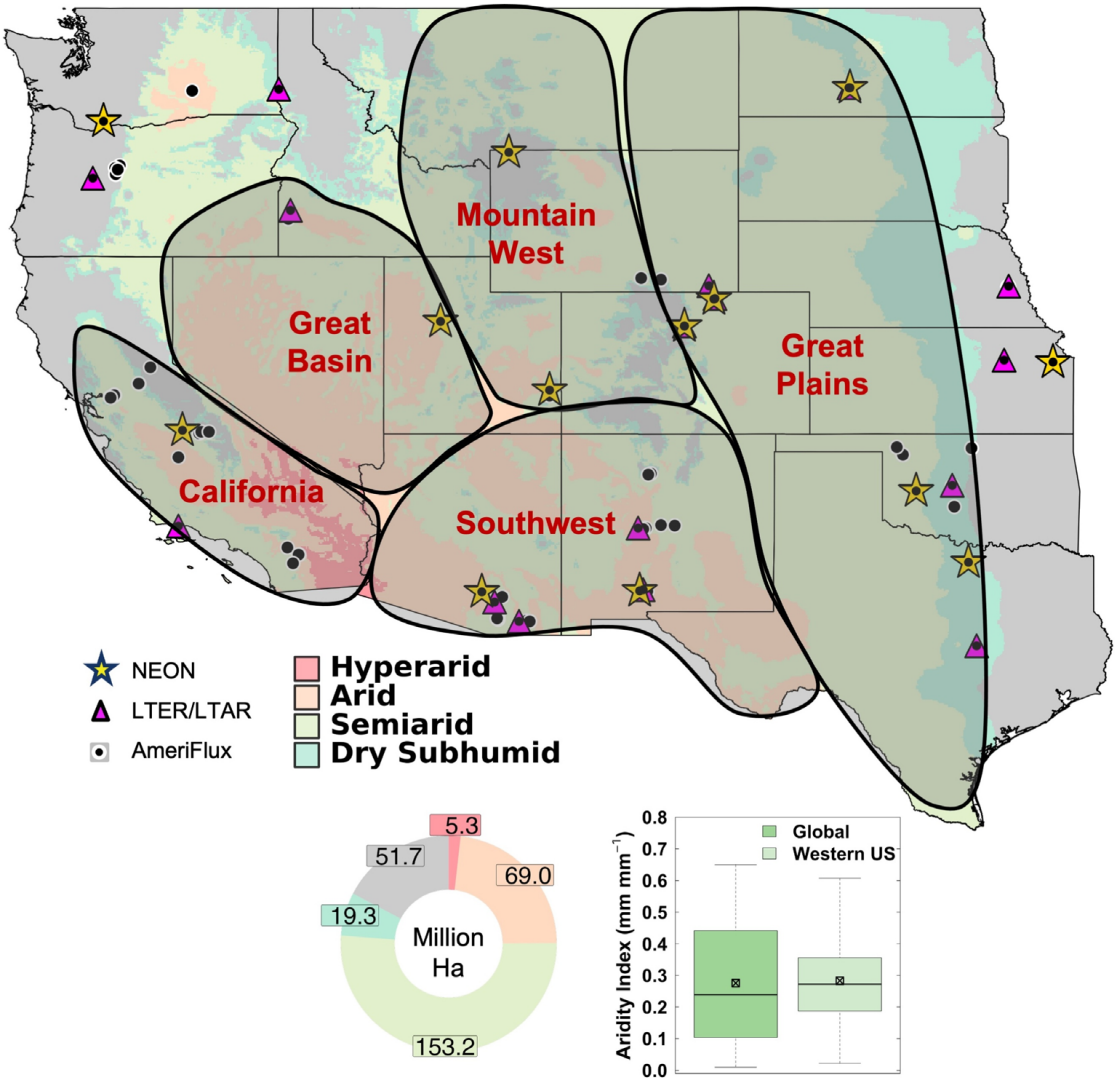
ARID's proposed intensive, threshold domain across the colored shading, with different colors representing dryland designations based on the aridity index. Focal areas are denoted with gray shading with approximate delineations within which more specific ARID implementations can be carried out (see Section 5.2.2. Focal Area Implementation: Intensive). Most of the region is arid and semi‐arid (pie‐chart) and its mean aridity is approximately the same as the mean aridity of global drylands (see boxplots). Map lines delineate study areas and do not necessarily depict accepted national boundaries.

The western U.S. meets the outlined criteria in having representative dryland features including a mean aridity index similar to that of the global dryland mean (Figure 5). It also notably represents diverse features of drylands including moisture distributions from arid to sub‐humid climates, different seasonalities of Mediterranean dry summers on the west coast to monsoon rainfall‐driven summers in the southwest, cold‐to‐hot gradients from north to south driven partly by elevation changes, and land cover gradients from rangelands throughout western U.S. eastward into croplands of the dry western half of the Great Plains. Additionally, this domain has extensive field networks, including AmeriFlux, NEON, and ARM sites (Figure 5), as well as many researcher and land manager personnel that would contribute to the campaign. The research community is also highly interested in the western U.S. drylands based on input across a variety of forums during our scoping, especially related to growing land management challenges under extreme drought and heat and limited water availability. Finally, conducting dryland field campaigns in the U.S. has several feasibility features including safety and high density of airports with feasible air traffic permissions.

Additionally, the ARID scoping team identified that, while there are extensive field networks and former aircraft measurements across the western U.S., these datasets have not been used substantially to advance understanding of dryland ecosystem processes of those labeled in Section 3. In fact, through working group discussions, the scoping team identified that the dryland research community was largely unaware of many former NASA aircraft campaigns. This creates an opportunity for multi‐temporal, multi‐sensor, strategically located airborne acquisitions that complement existing and on‐going efforts to address dryland science questions. Several examples of existing and ongoing aircraft campaigns to be leveraged for dryland science in the western U.S. includes but is not limited to the USGS's and NASA's Geological Earth Mapping Experiment (GEMx), NASA's Hyperspectral Infrared Imager (HyspIRI) and Western Diversity Time Series (WDTS), NASA's Goddard's LiDAR, Hyperspectral & Thermal Imager (G‐LiHT), NASA's FireSense, and NEON's Airborne Observation Platform (AOP) (Cook et al. 2013; Lee et al. 2015). Examples of existing field networks include but are not limited to AmeriFlux, NEON, and Long‐Term Agroecosystem or Ecological Research (LTAR/LTER).

#### 5.1.3 Distributed Domains

To increase science research representation of global drylands during the campaign, we propose several non‐U.S. study regions that would provide additional insight into drylands with different vegetation types, a wider range of hyperarid to subhumid conditions, different climatic forcing and variability, regions without strong snowmelt influence, among others. While we provide motivation for the selected distributed domains here, we emphasize that the dryland campaign can be carried out in other regions. The ultimate goal is to gain new insights beyond those available within the core western U.S. domain.

Substantial community interest was shown in conducting dryland research in southern African countries. Southern Africa includes gradients between hyperarid fog‐driven deserts and sub‐humid tropical woodlands, which is a wider climatic gradient than that observed in the western US. It also includes extensive university and non‐profit partners that are establishing measurement networks, conducting fundamental research, and studying sustainable land management practices.

Australia was also selected partly because it provides a key testbed for understanding dryland systems strongly driven by El Nino Southern Oscillation as well as its contribution to the interannual variability of the carbon cycle that contributes to ARID's science themes (Metz et al. 2023; Poulter et al. 2014). Australia also has strong in‐place research knowledge and measurement networks (OzFlux, TERN) with opportunities for co‐conducting field campaigns.

Our scoping activities also indicated strong community interest in research in northern Mexico given that it shares the Sonoran and Chihuahuan Deserts with the US. ARID scoping activities indicate potential remote sensing benefits for smallholder farming in this region as well as public agency interest in collaborating with dryland scientists on agricultural and rangeland applications.

Furthermore, community interest also motivated conducting dryland research in South America's Gran Chaco, Cerrado, and Caatinga (i.e., the South American Dry Diagonal). While containing mostly wetter domains, these areas are often experiencing drying and land use changes; an arid region was registered for the first time in northeast Brazil, the semi‐arid region is expanding at a rate of 75,000 km^2^ per decade (Tomasella et al. 2023), offering a testbed for understanding expansion of drylands. There are internationally driven research efforts a dryland field campaign can complement, including NeoTropTree, DryFlor, SECO, among others, which have established communities and measurement networks.

### 5.2 Campaign Implementation Strategy

#### 5.2.1 Overarching Implementation Strategies

Our implementation strategy in the ARID domains uses the following guiding principles: the need to address the dryland science questions in Section 3 and Table 1, multi‐temporal airborne acquisitions needed to address the science questions, scaling and development needs for remote sensing observations and modeling, leveraging existing assets (data and infrastructure) and particularly super sites, the safety and security of team members and affiliated scientists, optimal location of focal areas that bring the most science return on investment, and potential for co‐development with partners while being feasible and low risk. We expand on several overarching strategies here.

##### 5.2.1.1 Intensive and Distributed Sampling

It is not possible to sample the diverse range of global dryland landscapes and conditions in a field campaign under finite budgets. We propose intensive sampling, or more concentrated measurements in a smaller area, in a highly representative region (western U.S.) of global drylands while performing distributed sampling in other dryland regions with the help of partners and in‐place expertise to capture other features of drylands beyond what is available in the core domain.

##### 5.2.1.2 Spatial, Temporal, and Spectral Sampling With Multiple Sensors

Given the high spatial heterogeneity of drylands and rapidly changing conditions, the ARID field campaign strategy must accommodate sampling across spatial and temporal scales and spectra, which can be accomplished with aircraft sensors and augmented with drones, super sites, and former campaign data. These high spatial, temporal, and spectral scaling needs for drylands therefore must include coordinated use of in situ, drone, aircraft, and satellite measurements in the sampling strategy. The optimal field implementation approach in each of ARID's domains will depend on the emphasized science themes being addressed in those locations. For example, fine spatial sampling is necessary for vegetation structure and biodiversity themes, which require measurements at high resolution (< 1 m) to determine the degree to which larger spatial scales of satellites and models need to consider this sub‐pixel heterogeneity. High temporal sampling is necessary for pulse dynamics, drought, and fire themes, to characterize the rapid response of drylands to weather and climate. A range of spectral measurements is necessary for understanding interactions between themes (water‐carbon coupling), with microwave sensing providing information on water availability and carbon stocks, hyperspectral on ecosystem composition and soil processes, and lidar on ecosystem structure.

##### 5.2.1.3 ARID Super Sites Strategy

Many challenges of high spatiotemporal sampling needs of drylands can be overcome by leveraging super sites (Section 4.3.2). These sites can form the core locations of campaign airborne flights and drone surveys, providing collocation of field plots, instrumentation (e.g., flux towers and proximal sensing), and aircraft data. This integration is key for addressing complex dryland science priorities, including validating satellite retrievals, evaluating the full ecosystem response to pulse and drought dynamics, and testing remote sensing‐based retrieval approaches of vegetation structure or function parameters.

##### 5.2.1.4 Multi‐temporal Airborne Acquisition Strategy

Multi‐temporal sampling, or repeated airborne measurements over a specific area, was identified as a priority for gaining high spatial and high temporal resolution simultaneously to contextualize satellite observations. Multi‐temporal sampling strategies include (i) pulse chasing or making aircraft measurements in an area and waiting to re‐measure opportunistically immediately after a rain event (such on‐demand flight planning is possible in the U.S.), (ii) repeat flights during the evolution of an extreme event such as a drought or heatwave (iii) repeat seasonal sampling or re‐sampling of the same location in different phases of the growing season, and (iv) fusion approaches that create time series of a single aircraft overpass using drones resampling at a specific temporal cadence and/or with airborne acquisitions across ground sites with frequent sampling (super sites).

##### 5.2.1.5 Strategy to Leverage Manipulation Experiments

A dryland campaign can make use of high‐resolution remote sensing to observe in situ climate and land‐use experiments (i.e., DroughtNet, and Dragnet) and their controlled plot settings of background soil, climate, and atmospheric conditions. These field experiments manipulate environmental conditions, like excluding rainfall or altering rainfall patterns, to understand ecosystem responses. Combining remote sensing observations with known field responses can advance the ability to remotely sense, scale, and model these site responses to their environment and thus scale understanding of global change across larger dryland areas.

#### 5.2.2 Focal Area Implementation: Intensive

After overall domain selection, focal areas are preliminarily defined where campaign implementation can feasibly occur. The selection criteria for focal areas within the intensive domain (western U.S.) are as follows: ability to contribute to addressing ARID science themes, representation of diverse dryland environments and environmental gradients, locations that fill a specific knowledge gap, experiencing disturbances like the most pronounced extreme events and change, the existence of established field sites, co‐location with on‐going and/or former airborne campaigns, and containing priority land management locations identified by end‐users and practitioners.

Based on these criteria, we proposed focal areas in the intensive domain as the Southwest, Western Great Plains, Great Basin/Mojave Desert, Mountain West (defined here as Montana, Wyoming, Idaho, Utah, and Colorado), and California. It is anticipated that a multi‐year campaign can rotate between these focal regions, devoting all resources to each region over a set period. We provide brief motivation and example plans for each, while refraining from naming partners and institutions.

The Southwest focal area includes primarily Arizona and New Mexico and surrounding regions (Figure 5). Key features of this domain are extensive field sampling stations and strong in‐place knowledge by university, Tribal, and government‐based partners, which enable high potential to scale understanding of dryland processes between in situ, aircraft, and satellite scales. The region also has a southwest‐to‐northeast gradient of hot to cold conditions (with elevation gain) and transition from summer‐dominated to winter‐dominated seasonal precipitation. Key example studies include vegetation structure heterogeneity quantification, Tribal land management applications, water availability mapping, drought and fire disturbance studies, and pulse chasing of summer monsoon events (high frequency measurement campaigns after rainfall). For example, with strong summer monsoonal effects in the Southwest US, pulse chasing can be particularly useful for capturing vegetation and soil pulse responses to larger rainfall events. Both in situ field sampling teams and airborne teams can begin sampling after a large rainfall event to monitor both vegetation (pulse‐reserve) and soil (Birch effect) responses (Jarvis et al. 2007).

The Western Great Plains focal area extends between rangelands of eastern Colorado and croplands in the midwestern states in the domain, with the main goal of addressing land management questions. Example studies could include using remote sensing and ground measurements to identify plant functional types and quantify forage and crop yields for management decision tools. They also include more fundamental studies of using flux towers integrated with remote sensing to quantify grassland carbon uptake and east–west gradients in land‐atmosphere interactions.

The Great Basin and Mojave Desert focal area includes hyperarid deserts in Nevada and extending north, which is a notoriously under‐sampled area that is experiencing rapid increases in fire frequency, invasive grasses, and woody plant encroachment (Smith et al. 2023). Example campaign activities include using existing and new aircraft measurements as well as satellite remote measurements across spectra to identify invasive species, quantify wildfire impacts, and map minerals and biocrusts.

The Mountain West focal area consists of higher elevation, cold drylands across Wyoming, Montana, Idaho, Utah, and Colorado. Example campaign activities could include evaluating elevation and temperature gradients to determine the effects of lower temperatures on dryland vegetation function, differences in dryland vegetation structure at higher elevations, and effects of water sources (soil water vs. snowmelt) on ecosystem function.

The California focal area consists of hyperarid to arid in its southern portion and semi‐arid to dry subhumid (and Mediterranean) in its coastal to central region. Given extensive existing field sites and aircraft measurements, the region provides a testbed to begin early development of scaling or joint field‐aircraft methods to monitor biodiversity and wildfire hotspots (Chadwick et al. 2025; Harrison et al. 2024), while measurements are ongoing from other regions. The availability of aircraft data along aridity gradients provides opportunities to evaluate differences in phenologies, vegetation cover, and structure, as well as fire dynamics (Ward‐Baranyay et al. 2026).

#### 5.2.3 Focal Area Implementation: Distributed

The criteria for selection of focal areas in our distributed domain (non‐U.S.) are as follows: ability to expand the global dryland representativeness beyond the intensive western U.S. domain, contribution to ARID science questions from a global perspective, research experience and local knowledge of partners, compatible airborne assets with partners capable of providing airborne surveys if needed, availability of logistic support and infrastructure for fieldwork, and having high levels of safety for researchers during fieldwork.

We note that our distributed domain implementation strategy differs from our domestic strategy by aligning activities with existing, in‐place international research efforts in these focal areas that are addressing ARID's science themes. Aligning and co‐developing with in‐place research partners can help ensure continuity of the campaign activities with the assets and measurements being leveraged beyond the bounds of the ARID campaign and allow for leadership of in‐place researchers to champion the ARID campaign activities.

The range of partner research in each location tends to be broad. However, we anticipate several major research efforts in each region. Notably, studies in Australia are likely to contribute knowledge about interannual variability of the carbon cycle as well as effects of extreme temperatures on ecosystems. Research in southern Africa can focus on many topics including the contribution of savanna ecosystems to the carbon cycle, vegetation structure studies, sustainable land management of mainly smallholders, and wildfire research. Finally, South America studies can likely focus on effects of land use change from forest to agricultural uses as well as gradients of ecosystem function from wet to dry tropics.

### 5.3 Application Implementation: Data End‐User Support

A dryland field campaign should directly engage operational agencies (for the U.S., this includes BLM, USFS, USDA) and rural and Tribal communities, united around translating fundamental dryland science into improved decision making. For ARID, these partnerships began during scoping with their needs discussed and used to develop our science themes and implementation strategy (Feldman, Reed, et al. 2024). The ARID field campaign aims to improve the algorithm development and uncertainty estimation of fundamental remote sensing products that feed into existing workflows of agencies with established user‐basis and decision‐making frameworks. These applications can facilitate adaptation and mitigation to changes in drylands, demonstrating the societal value of dryland science to a large population of users.

Our suggested strategy to improve and facilitate end‐user decision making includes first understanding the end‐user needs and constraints. We have gained many insights about end‐user needs through over 160 engagement activities throughout 2023 and 2024 (Feldman, Reed, et al. 2024). However, ARID‐affiliated researchers will need to converse with the end‐users in each specific case. Boundary organizations (often public agencies and non‐profit organizations) can guide this process by bridging science and data end‐user needs. The next step is to develop the data product and/or monitoring tool and then iterate in discussion with the end‐user(s). For example, this can include developing invasive species cover or high spatial resolution water availability index maps with drone, aircraft, or satellite measurements and demonstrating or testing their usefulness in collaboration with the end‐user/decision maker, especially if ground observations are available for calibration and validation. In the final phase of implementing the new information into the decision‐making framework, the scientist needs to continue curating the data product as necessary and respond to any shortcomings identified by the end‐user. Such early, iterative, and continuous engagement with end‐users is critical to build the new knowledge and data products into the specific decision‐making frameworks. After an applied science project is carried out, impacts of the work can be quantified in terms of the end‐user's knowledge gain, extent of use, change in behavior, benefit, awareness and perception, and sustainability (St. Germain et al. 2024).

ARID envisions three types of application strategies to translate dryland measurements into social‐ecological systems applications that involve directly working and co‐developing with end‐users. The first type of strategy is a broader effort between a group of scientists and an organization that hosts application‐ready data products, like a public agency, where the group of scientists works to enhance an existing data product used by a wide array of users. For example, the USDA has applications like Rangeland Analysis Platform (RAP) that use algorithms to estimate vegetation type fractional cover and biomass from Landsat data to track rangeland conditions (Kleinhesselink et al. 2023; Vorster et al. 2025). A dryland field campaign, like ARID, has the potential to substantially improve the estimation of fine‐scale vegetation fractional cover and biomass through improved calibration and validation of algorithms trained with measurements from multi‐temporal hyperspectral airborne, UAS data, and satellite imagery. These improved fundamental input products can feed into the established workflows to inform widely used applications like RAP. Ultimately, such data enhancements more broadly improve information used for agricultural, invasive species, and wildfire management.

The second strategy is intensive efforts where smaller groups of scientists work directly with a decision maker or small decision maker group on their specific needs. Such an intensive, co‐development approach is needed for private ranchers and farmers and Tribal nations natural resources departments where developing trust is paramount. For example, both Tribal land managers and private ranchers in the southwestern U.S. defined priorities of monitoring water availability and viable rangeland forage on their specific lands. High spatial resolution datasets like microwave synthetic aperture radar (airborne or spaceborne) can be useful for evaluating distributions of surface soil moisture, while high resolution hyperspectral infrared measurements along with training data can be used to develop plant functional type identification methods for specific areas. While these efforts can be highly specific to fit an end user's need, the developed workflows can still be transferrable to other cases. The scientists will need to prioritize developing accessible, automated workflows that the end‐users can use long term.

A third strategy is for ARID‐based teams to work with boundary organizations (i.e., non‐profits, local universities) that engage with decision makers. Boundary personnel typically have long‐term existing relationships, domain knowledge, and communication experience, and can serve as liaisons between ARID‐affiliated researchers and decision makers. Therefore, ARID‐affiliated researchers can work together with a boundary organization that typically has an existing knowledge of local needs.

For U.S. domestic applications, the first two methods can be more suitable and efficient for U.S.‐based teams to carry out where cultural and geographic barriers between researcher and end‐user are typically reduced. Within the western U.S. intensive domain, opportunities were identified to work directly with practitioners, scientists, and decision makers at USFS, USGS, USDA ARS and NRCS, BLM, as well as private land managers (ranchers) on an array of activities including fire monitoring and fuel load estimation, invasive species mapping, rangeland forage productivity mapping, forest species mapping and drought response, soil moisture mapping, and others. Nevertheless, the third strategy is still applicable and can be used for domestic cases, especially when the boundary personnel have an established connection with an individual decision maker or decision‐making group.

For international applications, the third strategy is likely more appropriate where boundary organizations have existing long‐term relationships and understanding of needs and where geographic barriers and cultural differences between researcher and end‐user are prohibitive. A successful data‐to‐application process in this case hinges on careful and consistent iteration between the ARID‐affiliated researchers and the boundary organization as well as the boundary organization with the end‐users. Boundary organizations can be selected based on shared visions with ARID, such that boundary organization buy‐in is obtained via ARID enhancing an existing goal or process.

## 6 Future Perspectives

The ARID field campaign was borne out of substantial interest of the science and practitioner communities. These communities appear to be growing with increasing dryland focused publications (especially high impact) and well‐attended sessions dedicated to drylands at American conferences (American Geophysical Union and Ecological Society of America). The increasing community need and interest led to the NASA Terrestrial Ecology Program being selected to move forward as a scoping study in 2023. ARID scoping itself between 2023 and 2024 drew a large community to many events, resulting in over 160 engagement events with both intensive smaller workshop discussions between experts and broad audiences with sometimes over 200 people in attendance (Feldman, Reed, et al. 2024).

Such engagement events revealed that many fundamental questions remain unanswered as discussed in ARID's science agenda (Section 3; Table 1), partly prevented by deficient abilities to use remote sensing to detect dryland vegetation horizontal and vertical structure amongst background soil as well as errors in using remote sensing and modeling to quantify dryland water, carbon, and energy fluxes and their variations (Biederman et al. 2017; MacBean et al. 2021). These conversations and literature reviews also revealed that measurements in drylands were not always used to address these dryland science questions. Satellite‐based and model‐based soil, vegetation, and moisture information in drylands are ultimately in need of validation from these field measurements. These cross‐scale measurements also offer opportunities for new monitoring and prediction techniques. With only limited previous investments, there are thus opportunities for a more widespread dryland field campaign to address these critical and fundamental dryland science needs.

A dryland terrestrial field campaign would result in major scientific advancement in five core areas:

1. ARID can provide a substantially improved understanding of dryland contributions to global processes and how they respond to change. Drylands are understudied despite being globally extensive, with growing evidence showing they play a disproportionate role in the climate system. Joining remote sensing approaches with ground‐based measurements and monitoring, as well as with statistical and process‐based modeling and data analysis, can clarify the role of drylands in the Earth system.
2. The current generation of space‐based, aircraft, and UAS remote sensing sensors offers unprecedented potential to scale processes and services across areas that cannot be feasibly measured on the ground in heterogeneous drylands. ARID would advance our spatio‐temporal understanding of how drylands have already changed—including their growth into more mesic areas—and identify the main drivers that determine the size and direction of change.
3. ARID would directly support model development and evaluation. Earth system models are currently poor at capturing dryland drivers and responses to change, which drive large uncertainties in our understanding of global biogeochemical cycles and forecasts of future climate (Green et al. 2019; Simpson et al. 2024). ARID would directly address this critical knowledge gap by supplying the datasets needed to parameterize and evaluate dryland mechanisms, processes, and patterns across scales and into the future.
4. ARID offers an opportunity for calibration and validation of current and planned satellite missions. Given a relative lack of focus on drylands throughout satellite missions, there are large uncertainties in satellite retrievals of dryland geophysical properties. For example, retrievals across measurement frequencies are hindered by mixed bare soil, biocrust, and plant fractions, which create errors in retrievals of dryland infrared VIs and LST.
5. Remote sensing tools in drylands offer effective opportunities to support science‐informed decision making and for monitoring, evaluating, and prioritizing mitigation and adaptation solutions.

While focused on NASA and the United States, ARID serves as a blueprint for any future dryland field campaigns with both the science agenda and implementation strategy developed in intensive collaboration with the science community, public agencies, land managers, non‐profit organizations, and non‐U.S. entities. ARID's science themes are closely tied to numerous U.S. and international goals. Therefore, the new knowledge and datasets of such a dryland campaign would deliver are well‐aligned with the targets and information needs outlined by multiple U.S. and international policy efforts, which recognize the importance and high levels of change occurring in drylands, as well as the consequences for social‐ecological systems.

A dryland campaign could be used to address current and ongoing issues. ARID is capitalizing on a unique timing of increasing interest from end‐user parties and literacy with remote sensing data, as well as satellite remote sensing instruments increasingly becoming of higher spatial resolution (cm to 100 m scales) in both the public and commercial sectors (Figure 1). This includes agencies, like NASA, becoming more interested in applied sciences in rolling out plans to work more directly with end‐users under the Earth Science to Action Strategy (St. Germain et al. 2024). There is thus an opportunity for scientists to more directly apply their scientific knowledge and remote sensing processing capabilities for monitoring wildfire, water availability, invasive species, and crop and rangeland forage health.

In addition, increasingly extreme heat and drought events in drylands, with magnitudes and extents often outside the range of expectations, indicate that dryland campaigns can be beneficial now. Both science questions and social‐ecological systems research can help inform the adaptation and survival of humans and the ecosystems they live in, and remote sensing and on‐the‐ground information can substantially improve monitoring and decision‐making in these systems.

## Author Contributions

**Dennis Ojima:** conceptualization, writing – original draft, writing – review and editing. **Andrew F. Feldman:** writing – original draft, writing – review and editing, visualization, conceptualization, funding acquisition, project administration, supervision. **Joel A. Biederman:** writing – review and editing, conceptualization, funding acquisition. **Cibele Amaral:** writing – review and editing, conceptualization. **Russell L. Scott:** conceptualization, writing – review and editing, writing – original draft. **Niall P. Hanan:** conceptualization, writing – original draft, funding acquisition, writing – review and editing. **David J. P. Moore:** writing – review and editing, writing – original draft, conceptualization. **Alicja Babst‐Kostecka:** conceptualization, writing – review and editing. **Konrad Wessels:** writing – review and editing, writing – original draft, funding acquisition, conceptualization. **Natasha MacBean:** conceptualization, writing – review and editing, funding acquisition. **Sasha C. Reed:** funding acquisition, writing – original draft, writing – review and editing, visualization, conceptualization, supervision, project administration. **Flurin Babst:** conceptualization, writing – review and editing. **William K. Smith:** writing – original draft, writing – review and editing, funding acquisition, conceptualization. **Marcy Litvak:** conceptualization, writing – review and editing, writing – original draft. **Nikki Tulley:** conceptualization, writing – review and editing. **Julia K. Green:** conceptualization, writing – review and editing. **James Rattling Leaf Sr.:** conceptualization, writing – review and editing. **Ryan Pavlick:** writing – review and editing, project administration, conceptualization, supervision. **Raymond F. Kokaly:** writing – review and editing. **Shawn P. Serbin:** conceptualization, writing – review and editing. **Jennifer Watts:** writing – original draft, writing – review and editing, conceptualization. **Robert Swap:** conceptualization, writing – review and editing, writing – original draft, funding acquisition. **Julian Reyes:** writing – review and editing, conceptualization. **Compton J. Tucker:** conceptualization, writing – review and editing, funding acquisition. **Emily Kachergis:** conceptualization, writing – review and editing. **Lixin Wang:** conceptualization, writing – review and editing, writing – original draft. **Benjamin Poulter:** funding acquisition, conceptualization, writing – review and editing, writing – original draft. **Jasmine Ryan:** conceptualization, writing – review and editing. **Alejandro Flores:** conceptualization, writing – review and editing. **Karen Prentice:** writing – review and editing, conceptualization. **Tyson Swetnam:** conceptualization, writing – review and editing. **Robert A. Washington‐Allen:** conceptualization, writing – review and editing. **Henry W. Loescher:** conceptualization, writing – review and editing. **Michael D. SanClements:** conceptualization, writing – review and editing. **Allison K. Leidner:** writing – review and editing, conceptualization. **Sativa Cruz:** conceptualization, writing – review and editing.

## Funding

This work was supported by NASA Headquarters.

## Conflicts of Interest

The authors declare no conflicts of interest.

## Supporting information


**Table S1:** ARID research questions discussed throughout Section B.1. This table expands on Table 1 in showing more specific process level questions that can be addressed within each sub‐theme during the ARID campaign.
**Table S2:** ARID Science and Application Traceability Matrix shown only for select topics. The left column presents selected physical parameters required to address science sub‐themes (numbered in footnotes) and the remote sensing sensors available on proximal (e.g., flux towers), UAS, airborne, and spaceborne platforms. The names of the sensors are given in the footnotes of this table. The table lists the current and future sensors that can make the most significant advances and does not list all available options. Additionally, modeling solutions across different scales are discussed. Science Sub‐themes: 1.1 Water availability, 1.2 Dryland climate variability: Pulses and Drought, 1.3 Fire, 1.4 Land‐Atmosphere interactions, 2.1 Vegetation structure and Heterogeneity, 2.2 Biodiversity, 2.3. Ecosystem Function, 2.4 Dryland Geology and Soil Processes, 3.1 Carbon stocks and fluxes, 4.1 Land Management, 4.2 Adaptation and Mitigation.

## Data Availability

The dryland identification using the global aridity index was computed from TerraClimate data https://www.climatologylab.org/terraclimate.html. The aridity index maps and script to plot shading in figures 4 and 5 are available in Feldman and Smith (2024) in a Zenodo repository with DOI: https://doi.org/10.5281/zenodo.13371893.
