# Supplementary material for: Adaptation and Response in Drylands: A Dryland Research Agenda and Campaign Strategy
Source: Glob Chang Biol. 2026 Jul 15;32(7):e70975. doi: 10.1111/gcb.70975 (PMC13372540; doi:10.1111/gcb.70975)
Supplement: Supplementary file 1 — Table S1: ARID research questions discussed throughout Section B.1. This table expands on Table 1 in showing more specific process level questions that can be addressed within each sub‐theme during the ARID campaign. Table S2: ARID Science and Application Traceability Matrix shown only for select topics. The left column presents selected physical parameters required to address science sub‐themes (numbered in footnotes) and the remote sensing sensors available on proximal (e.g., flux towers), UAS, airborne, and spaceborne platforms. The names of the sensors are given in the footnotes of this table. The table lists the current and future sensors that can make the most significant advances and does not list all available options. Additionally, modeling solutions across different scales are discussed. Science Sub‐themes: 1.1 Water availability, 1.2 Dryland climate variability: Pulses and Drought, 1.3 Fire, 1.4 Land‐Atmosphere interactions, 2.1 Vegetation structure and Heterogeneity, 2.2 Biodiversity, 2.3. Ecosystem Function, 2.4 Dryland Geology and Soil Processes, 3.1 Carbon stocks and fluxes, 4.1 Land Management, 4.2 Adaptation and Mitigation. [file GCB-32-e70975-s001.docx]

*Table S1. ARID research questions discussed throughout Section B.1. This table expands on Table 1 in showing more specific process level questions that can be addressed within each sub-theme during the ARID campaign.*

| **Research Theme** | **Theme Questions** | |
| --- | --- | --- |
| **Theme 1: Climate Variability and Drought** | ***How are extremes like droughts, heatwaves, and large rain pulses across drylands impacting water availability, and are these extremes amplified by changing fire***  ***regimes, land cover change, and land-atmosphere interactions*?** | |
| **Sub-Theme** | **Central Sub-Theme Question** | **Potential Process-level Questions** |
| **1.1 Water Availability** | How do changes in the amount, timing, intensity, and phase of water inputs affect surface water partitioning among evaporation, transpiration, runoff, and groundwater recharge, thereby regulating the amount of water that is available to humans and ecosystems? | How do changes in atmospheric conditions, such as CO2, VPD, and energy balance, alter the fate and temporal variability of soil moisture?  How can remote sensing detect, and Earth System Models predict, key physical parameters (e.g., precipitation, evapotranspiration, soil moisture, plant water status, and runoff) to address these questions? |
| **1.2 Dryland Climate Variability: Pulses and Droughts** | How do dryland ecosystems process heterogeneous, highly dynamic moisture pulses?  How does the timing and duration of lack of rainfall impact dryland function, structure, composition, and water availability?  How is drought intensity, severity, and duration changing in drylands? | How are moisture pulses changing?  How can Earth observation adequately capture pulse dynamics at the appropriate diurnal and daily-scale frequency?  Do process models capture rain/non-rainfall pulses and ecosystem responses?  How do higher temperatures and heatwaves influence drought onset and duration in drylands?  Which dryland ecosystem components respond most to drought? |
| **1.3 Fire** | How do fire regimes on both rangeland, open woodland, and forested landscapes change the composition, structure, and function of drylands at various time scales? | How do changes in dryland composition, structure, and function feed back to impact fire regimes?  What role does fire play in the expansion of invasive species, shrub encroachment, and the loss of ecosystem services as observed across multiple continents?  How does fire impact carbon stocks and fluxes across diverse dryland ecosystem types and across spatio-temporal scales? |
| **1.4 Land-atmosphere Interactions** | How do land-atmosphere interactions influence climate extremes, water availability, and dryland ecosystem responses, including changes in air temperature, changes in the frequency and intensity of extreme events, as well as land use change? | To what degree are land-atmosphere interactions driving drylands’ contribution to water, carbon, and energy fluxes?  How much are climate extremes playing a role in these interactions?  How does dryland heterogeneity of soil conditions (e.g., soil moisture, soil texture) and vegetation (structure and types) influence landscape-scale land-atmosphere interactions (like convection)? |
| **Theme 2: Ecosystem Structure, Function, and Biodiversity** | ***What are the main mechanisms driving the spatiotemporal distributions of dryland structure, function, and biodiversity?*** | |
| **Sub-Theme** | **Central Sub-Theme Question** | **Select Process-level Questions** |
| **2.1 Vegetation Structure and Heterogeneity** | How are ongoing changes in climate (rising CO2, increases in drought frequency and intensity, long-term changes in mean rainfall, and changing fire regimes) and land use (changing fire regimes, grazing, and other land uses) impacting vegetation structure, function, and habitat in drylands? | What are the rates and underlying causes of woody plant encroachment (WPE) in global drylands?  How do invasions by exotic shrubs and grasses observed in drylands around the world impact ecosystem function and disturbance regimes?  How are changes in structure and function impacting essential ecosystem services, including changes in water, carbon, and nutrient cycling in drylands and the provision of forage for livestock? |
| **2.2 Dryland Biodiversity** | What are the drivers of biodiversity (functional, phylogenetic, taxonomic) in drylands, and how will these be changing? | What is the relationship between biodiversity, ecosystem function, carbon stocks, and resilience to disturbances (drought and fire)?  What are the impacts of increased aridity and continued grazing pressure on biodiversity (functional, phylogenetic, taxonomic, etc.)?  What is the impact of land cover transformation, land degradation, and cultivation on dryland biodiversity and species of special interest (e.g., endangered species)?  What are the impacts of invasive plant species on biodiversity, rangeland conditions, and water availability? |
| **2.3 Ecosystem Function** | Across different timescales, what are the dominant mechanisms driving dryland function, such as plant hydraulics, leaf-level photosynthesis, respiration, and nutrient cycling? | What role has CO2 fertilization played in driving changes in the SPAC and dryland functions GPP, NEE, ET, and WUE?  What roles have the timing and intensity of precipitation and temperature-driven increases in VPD played in driving changes in the SPAC and dryland functions GPP, NEE, ET, and WUE?  Can we utilize remote sensing data to improve the estimation of dryland vegetation physiological status (e.g., photosynthetic quantum yield, nutrient, pigment, and enzyme concentrations, canopy stomatal and hydraulic properties, vegetation and soil water status, drought stress) for distinct PFT and biocrust communities?  Can we improve retrievals of vegetation function (e.g., carbon, water, and energy fluxes) in drylands, either directly (e.g. thermal estimation of energy balance), or via the use of improved structural, physiological, and phenological retrievals in process-based models?  Can improvements of decomposition of soil organic matter be improved by night-time monitoring of carbon exchanges and soil moisture levels? |
| **2.4 Dryland Geology and Soils** | How do soil communities and physicochemical characteristics drive and respond to climate variability and ecosystem change? | What is the extent, composition, and function of dryland biological soil crusts (biocrusts)?  What roles do soils play in dictating aboveground structure, function, and response to change?  How much inorganic carbon is stored in dryland soils across different soil types, aridity index gradients, and natural vs. managed systems, and how vulnerable is the carbon to environmental shifts?  What contribution do soil signals play in space-based observations of all terrestrial ecosystems?  What are the sources, sinks, causes, and consequences of accelerated topsoil loss and dust in Earth’s drylands? |
| **Theme 3: Carbon Cycle Interannual Variability and Long-Term Trends** | ***What is the contribution of drylands to the mean, trend, and particularly the interannual variability of terrestrial carbon dynamics?*** | |
| **Sub-Theme** | **Central Sub-Theme Question** | **Select Process-level Questions** |
| **3.1 Carbon Stocks and Fluxes** | How large are the carbon stocks and fluxes in drylands, how do they vary at sub-annual to decadal timescales, and what is their response through space and time to drivers of global change? | How vulnerable are dryland carbon stocks and fluxes to global and regional changes in water availability and atmospheric demand, combined with asynchronous plant responses to periods of water stress?  How do woody encroachment and desertification change dryland carbon cycling and feedbacks?  What is the relative contribution of different plant functional types to carbon stocks, and how are these changing along environmental gradients?  What influence do rooting strategies and belowground carbon allocation play in ecosystem carbon storage?  What is the potential of carbon capture in dryland vegetation and soils to work as a nature-based climate solution (NbCS)?  How can remote and in-situ observations be best used to model and quantify carbon stocks and fluxes? |
| **Theme 4: Social Ecological Systems** | ***What are the consequences of changes in drylands for social-ecological systems and what management (e.g., mitigation and adaptation) solutions can maintain the critical services provided by drylands even in the face of change?*** | |
| **Sub-Theme** | **Central Sub-Theme Question** | **Select Process-level Questions** |
| **4.1 Land Management** | How are land and water resources and resource management being affected by drought and aridity changes? | How are the composition and productivity of rangelands changing under various management regimes and how are they predicted to change?  How will remote sensing and modeling enhance assessment and forecasting of land resources (e.g., soil moisture, productivity, water use, carbon storage) for various land uses?  How will land use further impair wildlife corridors and migratory pathways?  How will further fragmentation of conservation and rangeland areas affect biodiversity and conservation goals?  How will reduced water availability impact forage production, cropping yields, livestock conditions, and habitat conditions?  How will increased thermal stress affect agricultural production of rangelands, cropland, forests, livestock, wildlife, and wildland systems?  How will renewable energy deployment affect ecosystem services?  Can multi-sensor observations and modeling improve our ability to assess land management effectiveness? |
| **4.2 Adaptation and Mitigation Strategies** | ***Adaptation and resilience of dryland social ecological systems***  How can dryland ecosystems enhance their resilience in the face of environmental stressors, and what adaptive strategies can local communities implement to build resilience?    ***Mitigation options for different dryland livelihood strategies***  How will climate variability and water limitations hinder carbon sequestration efforts? | ***Adaptation and resilience of dryland social ecological systems***  What factors and information can enhance the adaptive capacity of dryland social-ecological systems to meet the challenges of increased aridity?  How can ecological forecasting of drought conditions enable decision-making of dryland operators and natural resource managers?  Have land managers needed to change their strategies in drylands in the past few decades to accommodate change? What drives these changes?  ***Mitigation options for different dryland livelihood strategies***  What impact will renewable energy development and carbon sequestration practices have on dryland livelihood strategies and ecosystem services in different dryland regions?  How can improved observations enhance our assessment of renewable energy systems and carbon sequestration practices?  What increased information can be provided related to changes in ecosystem processes and ecosystem services related to mitigation practices? |

***Table S2.*** *ARID Science and Application Traceability Matrix shown only for select topics. The left column presents selected physical parameters required to address science sub-themes (numbered in footnotes) and the remote sensing sensors available on proximal (e.g., flux towers), UAS, airborne, and spaceborne platforms. The names of the sensors are given in the footnotes of this table. The table lists the current and future sensors that can make the most significant advances and does not list all available options. Additionally, modeling solutions across different scales are discussed. Science Sub-themes: 1.1 Water availability, 1.2 Dryland climate variability: Pulses and Drought, 1.3 Fire, 1.4 Land-Atmosphere interactions, 2.1 Vegetation structure and Heterogeneity, 2.2 Biodiversity, 2.3. Ecosystem Function, 2.4 Dryland Geology and Soil Processes, 3.1 Carbon stocks and fluxes, 4.1 Land Management, 4.2 Adaptation and Mitigation.*

| **Physical Parameter or Process/ Observable** | **Science theme number** | **Comment: Need and Current Status** | **Ground-based and Proximal Remote Sensing** | **UAS Remote Sensing** | **Airborne Remote Sensing** | **Spaceborne/**  **Satellite Remote Sensing** | **Land Surface Modeling** |
| --- | --- | --- | --- | --- | --- | --- | --- |
| Soil Moisture  (SM) | All | Key driver for local models and validation/diagnostic for the water cycle in LSMs/ESMs.  Poorly estimated for the subsurface and root zone; Need for higher resolution (10 m to <1 km) estimates. | Soil moisture network, GNSS receiver (interferometric reflectometry), microwave radiometers, cosmic ray neutron sensing | Microwave radiometers, Thermal cameras:  FLIR A700f; ICI P-Series;  Micasense | UAVSAR, SLAP, AirMoss, SMAPVEX; NOAA airborne gamma NOHRSC, AirSWOT, | SMAP, ECOSTRESS, Hydrosat, SBG, SMOS, NISAR, Sentinel | Evaluate soil moisture responses to rainfall pulses and drydowns, increased evaporative demand, and seasonal to inter-annual variations in rainfall. Drive modeled soil nutrient dynamics. A key input to many landscape and vegetation distribution models. |
| Evapotranspiration (ET) | All | Need for calibration/validation for model estimates of ET, E, and T. OpenET models can use multiple RS inputs. | Infrared Thermometer (IRT): Apogee SI-111-SS  Thermal Camera: FLIR A700f;  ICI P-Series;  Micasense | Thermal Cameras:  FLIR A700f; ICI P-Series;  Micasense | HYTES, MASTER, G-LiHT | ECOSTRESS, Landsat,  Sentinel, Hydrosat, SBG | Calibration/validation needed for modeled ET. Evaluate modeled plant transpiration vs bare soil evaporation across timescales |
| Surface temperature (ST) of soil vs. plants | All | Input into ET, GPP, NEE, and vegetation stress algorithms. | Infrared Thermometer (IRT): Apogee SI-111-SS  Thermal Camera: FLIR A700f;  ICI P-Series; Micasense | Thermal  Cameras:  FLIR A700f; ICI P-Series;  Micasense | HYTES, MASTER, G-LiHT | ECOSTRESS, Landsat,  Sentinel, VIIRS, GOES, Hydrosat, SBG | Evaluate modeled surface energy balance and evaluate use as an input variable for ET models and intermediate complexity carbon cycle models. |
| Vegetation fractional cover of plant functional types (PFT) and biocrusts | All | Need for partitioned high resolution mapping of trees, shrubs, grasses, forbs, biocrust.    Local calibration/ initialization needed for models. | PhenoCam; Terrestrial Laser Scanning (TLS) | Various multi-spectral Cameras;  Various lidar instruments | AVIRIS-NG, NEON-AOP, G-LiHT, LVIS | Landsat, Sentinel, SBG, EMIT, PACE | Initialize mixed grass-shrub cover and type; evaluate prognostic vegetation changes in dynamic vegetation models. Serves as an input variable for ecosystem models and LSMs |
| Vegetation structure (height and canopy cover) | 1.3, 1.4, 2.1, 2.2, 3.1 | Shrub (<3m) biomass poorly estimated with optical and space-based lidar algorithms. Individual tree heights can be extracted using structure from motion (SfM) from airborne imagery. Potential constraint for data assimilation | Terrestrial Laser Scanning (TLS) | High resolution cameras and structure from motion (SfM) photogrammetry; Various lidar instruments | Discrete Lidar, G-LiHT,  LVIS, UAVSAR, AVIRIS-NG, NEON-AOP | NISAR, Sentinel, GEDI, IceSat2, WorldView, PlanetScope | Initialise tree/shrub height; evaluate prognostic vegetation changes in dynamic vegetation models. Provides PFT information for parameterizing ecosystem models and grid heterogeneity of LSMs. |
| Above ground biomass: grass | 1.3, 2.1, 2.3, 2.2, 3.1, 4.1, 4.2 | Grass biomass poorly estimated with optical; useful for calibration/validation of ecosystem models | Terrestrial Laser Scanning (TLS) | Various multi-spectral cameras;  Various lidar instruments | AVIRIS-NG, G-LiHT, NEON-AOP | EMIT, PACE, Landsat, Sentinel, SBG, SMAP | Evaluate aboveground biomass and forage amounts, which helps to constrain belowground C stocks, estimate GPP, respiration, and forage yields |
| Above ground biomass: shrubs and trees | 1.3, 2.1, 2.2, 2.3, 3.1, 4.1, 4.2 | Calibration/Validation for model estimates of NPP.  Potential constraint for data assimilation. Shrub biomass is poorly estimated with optical and space-based LiDAR algorithms. | Terrestrial Laser Scanning (TLS) | Various multi-spectral cameras;  Various lidar instruments | Discrete Lidar, G-LiHT  LVIS, UAVSAR, AVIRIS-NG, NEON-AOP | NISAR, Sentinel, GEDI, IceSat2, | Provides estimates of woody contribution to aboveground biomass and landscape distribution of browse and forage availability. Helps constrain belowground C stocks. Input information for partitioning GPP of dryland landscapes. |
| Photosynthetically active vegetation and plant traits | 1.2, 1.3, 2.3, 3.1 | Essential to NPP algorithms based on light use efficiency approach. Active and non-active components; canopy nitrogen content; canopy chlorophyll content; canopy xanthophyll cycle conversion | Phenocams,  Multispectral cameras and sensors; spectrometers | Various multi-spectral cameras; Various hyperspectral instruments | AVIRIS-NG, LVIS, G-LiHT | EMIT, PACE, EnMAP, SBG, Landsat, Sentinel | Develop new dryland plant grass and drought-deciduous phenology schemes and constrain associated parameters. Enables partitioning of live and dead biomass plus fraction of dominant leaf biomass of dryland landscapes. Enables new constraints related to nutrient availability and photoprotection. |
| Vegetation moisture content, live fuel moisture content | 1.1, 1.2, 1.3, 1.4, 2.3, 4.1 | Can be used to build pressure-volume curves to estimate plant water potential. Multi-spectral approaches only capture canopy water content. | TLS; microwave radiometers; GNSS transmissometry (GNSS-T) for taller vegetation, GNSS interferometric reflectometry (GNSS-IR) for shorter vegetation | microwave radiometers; Various hyperspectral instruments | AVIRIS-NG, UAVSAR | Sentinel, SMAP, NISAR, EMIT, EnMAP, SBG, Landsat, HLS, PACE | Constrain parameters of plant hydraulics schemes and live fuel moisture content as well as evaluate their responses to changes in surface temperatures, water availability pulses, and periods of water stress |
| Invasive species: tree, grass, shrub, forbs | 1.3, 2.1, 2.2, 4.1, 4.2 | Individual invasive plant species could be spectrally distinguished or indirectly distinguished with phenology approaches | Hyperspectral: ASD FieldSpec; Spectra Evolution RS-3500, 8800, PSR+ | Various multi-spectral cameras; Various hyperspectral instruments | AVIRIS-NG, G-LiHT | EMIT, EnMAP, SBG, Landsat | Provide spatial information on species distribution of landscape models |
| Solar-induced Fluorescence (SIF) | 1.2, 1.4, 2.3, 3.1 | A means to improve GPP estimates in remote sensing and model products | Ultra-hyperspectral VNIR: PhotoSpec; FluoroSpec; FLOX Box | Ultra-hyperspectral VNIR instruments | FIREFLY, CFIS | OCO-2,3, TEMPO, TROPOMI | Evaluate and constrain photosynthesis across timescales, especially during periods of heat and water stress |
| Fraction of photosynthetically active radiation absorbed (FAPAR) per PFT | 2.3, 3.1 | Input to carbon-flux models | Hyperspectral, Multi-spectral | Various multi-spectral cameras; Various hyperspectral instruments | AVIRIS-NG, G-LiHT | EMIT, EnMAP, SBG, GOES-R | Evaluate and constrain phenology and GPP |
| Gross and Net Primary Production: Trees, grass, shrubs. | 1.2, 1.4, 2.3, 3.1, 4.2 | Modeled variables that require FAPAR of PFT and meteorological variables as input. SIF could provide a dynamic estimate of light use efficiency (LUE). | Hyperspectral; Thermal; SIF | Various multi-spectral cameras; Various thermal cameras; Various hyperspectral instruments | AVIRIS-NG, G-LiHT, FIREFLY, CSIF | OCO-2,3, PACE, SBG, GOES-R, Landsat, Sentinel | Evaluate and constrain parameters of ecosystem-scale C fluxes. Improved representation at the PFT-level. Improved representation of plant physiology using novel metrics such as canopy temperature and SIF. |
